# Preeclampsia-Associated Alteration of DNA Methylation in Fetal Endothelial Progenitor Cells

**DOI:** 10.3389/fcell.2019.00032

**Published:** 2019-03-19

**Authors:** Lars Brodowski, Tristan Zindler, Sandra von Hardenberg, Bianca Schröder-Heurich, Constantin S. von Kaisenberg, Helge Frieling, Carl A. Hubel, Thilo Dörk, Frauke von Versen-Höynck

**Affiliations:** ^1^Department of Obstetrics and Gynecology, Hannover Medical School, Hanover, Germany; ^2^Psychiatry, Social Psychiatry and Psychotherapy, Hannover Medical School, Hanover, Germany; ^3^Department of Obstetrics, Gynecology, and Reproductive Sciences, Magee-Womens Research Institute, University of Pittsburgh School of Medicine, Pittsburgh, PA, United States

**Keywords:** preeclampsia, DNA methylation, epigenetics, endothelial colony-forming cells, fetal programming

## Abstract

**Objective:**

The pregnancy complication preeclampsia represents an independent risk factor for cardiovascular disease. Our previous research shows a diminished function of fetal endothelial colony-forming cells (ECFC), a proliferative subgroup of endothelial progenitor cells (EPC) in preeclampsia. The aim of this study was to further investigate whether DNA methylation of fetal EPC is affected in preeclampsia.

**Methods:**

The genomic methylation pattern of fetal ECFC from uncomplicated and preeclamptic pregnancies was compared for 865918 CpG sites, and genes were classified into gene networks. Low and advanced cell culture passages were compared to explore whether expansion of fetal ECFC in cell culture leads to changes in global methylation status and if methylation characteristics in preeclampsia are maintained with increasing passage.

**Results:**

A differential methylation pattern of fetal ECFC from preeclampsia compared to uncomplicated pregnancy was detected for a total of 1266 CpG sites in passage 3, and for 2362 sites in passage 5. Key features of primary networks implicated by methylation differences included cell metabolism, cell cycle and transcription and, more specifically, genes involved in cell-cell interaction and Wnt signaling. We identified an overlap between differentially regulated pathways in preeclampsia and cardiovascular system development and function. Cell culture passages 3 and 5 showed similar gene network profiles, and 1260 out of 1266 preeclampsia-associated methylation changes detected in passage 3 were confirmed in passage 5.

**Conclusion:**

Methylation modification caused by preeclampsia is stable and detectable even in higher cell culture passages. An epigenetically modified endothelial precursor may influence both normal morphogenesis and postnatal vascular repair capacity. Further studies on epigenetic modifications in complicated pregnancies are needed to facilitate development of EPC based therapies for cardiovascular alterations.

## Introduction

The hypertensive pregnancy disorder preeclampsia is one of the most common causes of maternal and fetal morbidity and mortality in the developed world (Roberts, 2000; Wang et al., 2004). Impaired placental development early in pregnancy and consequent release of several placenta-derived factors into the maternal circulation is thought to lead to generalized maternal endothelial dysfunction as a main clinical feature in the second half of pregnancy (Cockell et al., 1997; Maynard et al., 2003; Redman and Sargent, 2005).

Recent studies suggest that preeclampsia not only represents an independent, cardiovascular risk factor for the mother, but that the cardiovascular system of the offspring is also adversely affected. The affected children display on average significantly higher blood pressure, higher body mass index (BMI) and increased vascular stiffness in the pulmonary and peripheral circulation compared to offspring from uncomplicated pregnancies (Jayet et al., 2010; Davis et al., 2012). Epidemiological studies suggest that cardiovascular disease can originate during fetal development (Barker et al., 1989). The exact molecular mechanisms are still unclear, but epigenetic changes may be involved.

Endothelial progenitor cells (EPC) play an important role in vascular homeostasis and development, including repair of injured endothelium, angiogenesis, and neovascularization (Murayama and Asahara, 2002). A decreased cell number of early outgrowth, hematopoietic EPCs in the maternal circulation has been described as a potential sign of impaired endothelial repair capacity in preeclampsia (Lin et al., 2009; Luppi et al., 2010). We previously found lower numbers and impaired function of fetal endothelial colony forming cells (ECFC), a late outgrowth sub-class of EPC, in pregnancies complicated by preeclampsia. ECFCs are a relatively homogeneous cell population able to proliferate and migrate to sites of vessel formation where they are directly involved in building the endothelium (Ingram et al., 2004; Urbich and Dimmeler, 2004). They are critical to, and play a complementary role with other EPCs, in blood vessel formation and repair (Sipos et al., 2010), and they have been implicated as biomarkers for cardiovascular risk (Dignat-George et al., 2003; Ingram et al., 2017).

Studies on epigenetic modifications in complicated pregnancies are urgently needed to develop epigenetically based therapeutics for the prevention and therapy of early cardiovascular alterations. Considering the potential of cell therapies based on EPCs, the aims of this study were to compare the DNA methylation status of fetal ECFCs at birth between uncomplicated and preeclamptic pregnancies, and to assess the effect of cell culture on DNA methylation.

## Materials and Methods

### Patients

The Ethical Committee at Hannover Medical School and the University of Pittsburgh Institutional Review Board approved the study. Informed written consent was obtained from each study participant. Twelve healthy women with uncomplicated, normotensive pregnancies (controls) and 12 women with late-onset preeclampsia (diagnosis at >37 weeks gestation) provided umbilical cord blood samples directly after delivery. Clinical and demographic data of these women are presented in Table 1. All had singleton pregnancies. Patients were frequency (group) matched for gestational age at the time of delivery, BMI and race. Patients with preeclampsia had gestational hypertension and proteinuria beginning after 20 weeks of pregnancy with resolution of clinical symptoms postpartum (ACOG Committee on Practice Bulletins–Obstetrics, 2002). Gestational hypertension was recognized as an absolute blood pressure ≥ 140 mmHg systolic and/or ≥ 90 mmHg diastolic after 20 weeks of gestation. Proteinuria was defined as ≥ 300 mg per 24h urine collection, ≥ 2+ protein on a voided urine sample, ≥ 1+ protein on a catheterized urine specimen, or a protein-creatinine ratio of ≥0.3. Women with uncomplicated pregnancies were normotensive and without proteinuria throughout gestation, and delivered healthy babies at term. All patients were without clinical history of preexisting renal, vascular, or metabolic disease.

**Table 1:** Patient demographics.

	Uncomplicated pregnancy (*n* = 12)	Preeclamptic pregnancy (*n* = 12)	*p* value
Maternal age (years)	29.3 ± 6.1	28.6 ± 5.6	0.76
Gestational age at delivery (weeks)	39.0 ± 0.7	37.7 ± 2.0	0.06
Multiparous *n* (%)	7 (58%)	8 (67%)	1.0
Maternal pre-pregnancy BMI (kg/m^2^)	25.4 ± 5.1	27.8 ± 9.8	0.47
Gestational SBP, pre-delivery (mmHg)	122 ± 9.6	153 ± 19.5	**<0.0001^∗^**
Gestational SBP, before 20 week gestation (mmHg)	114 ± 13.6	122 ± 15.8	0.18
Gestational DBP, pre-delivery (mmHg)	73.4 ± 8.2	93.6 ± 9.8	**<0.0001^∗^**
Gestational DBP, before 20 week gestation (mmHg)	70.4 ± 10.5	76.5 ± 10.2	0.17
Birth weight (g)	3467 ± 417	2848 ± 645	**0.01^∗^**
Birth weight percentile	49.2 ± 28.5	29.8 ± 26.2	0.1
Birth weight percentile < 10th, *n* (%)	0 (0%)	2 (16.3%)	0.48
Cesarean delivery *n* (%)	9 (75%)	7 (58%)	0.42
Maternal race, White *n* (%)	10 (83%)	9 (75%)	1.0
Baby sex, Male *n* (%)	9 (75%)	5 (42%)	0.11

*Data are expressed as mean ± standard deviation or number (*n*) and %. Distribution was examined with Kolmogorov–Smirnov test. Continuous data were compared with unpaired *t*-test or Mann–Whitney test, as appropriate. Categorical variables were compared by Fisher’s exact test. BMI body-mass index; SBP systolic blood pressure; DBP diastolic blood pressure.*

### ECFC Isolation

Endothelial colony-forming cells isolation and characterization was performed following previously published protocols (Grundmann et al., 2012; Brodowski et al., 2014). Briefly, peripheral blood mononuclear cells (PBMCs) were isolated from 40 to 50 mL of venous cord blood by gradient centrifugation (1500 rpm, Ficoll Plus, GE Healthcare, Buckinghamshire, England or Piscataway, NJ). The washed PBMCs were plated (5 × 10^7^ cells/well) onto collagen-coated 6-well plates (BD Bioscience, Heidelberg, Germany or Billerica, MA), containing endothelial growth medium-2 (EGM-2, Lonza, Basel, Switzerland) supplemented with suppliers recommended concentrations of growth factors, 10% FBS and 1% penicillin/streptomycin. Growth media were changed daily for the first seven days and then every other day. Colonies were assessed daily, and colony numbers and time of colony appearance were recorded. Colonies were then clonally expanded and banked for the studies as described below. The phenotype of each ECFC clonal line was confirmed by its “cobblestone” monolayer morphology, determination of acetylated low-density lipoprotein uptake and Ulex europaeus lectin binding, capillary tubule-like network formation in Matrigel assay, expression of characteristic surface markers (CD31, CD34, KDR) and the absence of hematopoietic or myeloid surface antigens (CD45, CD14 and CD133), as previously described (Brodowski et al., 2014).

### DNA Sample Preparation

Endothelial colony-forming cells at 80–90% confluence were extensively rinsed in cold PBS, mechanically scraped from the flask, snap frozen and stored at –80°C. Further processing was continued for all samples simultaneously. First, 1–5 mg of cells were homogenized using QIAshredder columns (Qiagen, Leipzig, Germany) and genomic DNA and total RNA were isolated from the cell lysates using the AllPrep DNA/RNA micro kit (Qiagen, Leipzig, Germany).

### Methylation Analysis

DNA samples were subjected to methylation analyses using the Illumina Infinium EPIC BeadChip Kit (Illumina, San Diego, CA, United States) at the University of Kiel, Institute of Clinical Molecular Biology (ICMB). Native DNA was sent from our lab to ICMB. The samples were coded for blinded sample analysis by ICMB personnel. The assay required 200 ng of original DNA sample as input. The DNA samples were denatured and neutralized and were then isothermally amplified in an overnight step. The whole-genome amplification uniformly increased the amount of the DNA sample by several thousand-fold without significant amplification bias. A controlled enzymatic process fragmented the amplified product. The process used endpoint fragmentation to prevent overfragmentation. The fragmented DNA was collected after an isopropanol precipitation and centrifugation at 4°C. The precipitated DNA was resuspended in hybridization buffer. Samples were applied to a BeadChip and separated by an IntelliHyb seal. The loaded BeadChip was incubated overnight in an Illumina Hybridization Oven. The amplified and fragmented DNA samples annealed to locus-specific 50-mers during hybridization. Unhybridized and non-specifically hybridized DNA was washed off and the BeadChip was prepared for staining and extension. Single-base extension of the oligos on the BeadChip, using the captured DNA as a template, incorporated detectable labels on the BeadChip and determined the genotype call for the sample. XStain occurred in a capillary flow-through chamber. The Illumina HiScan System scanned the BeadChip, using a laser to excite the fluorophore of the single-base extension product on the beads. The scanner recorded high- resolution images of the light emitted from the fluorophores. Processed data were sent back to our lab for analysis.

### Network and Pathway Analysis

We compared methylated genes from preeclampsia versus uncomplicated pregnancy at passage 3 or passage 5 using the database of the Gene Ontology (GO) Consortium ^1^ (Gene Ontology Consortium, 2015) for an enrichment of biological pathways as defined in the Kyoto Encyclopedia of Genes and Genomes (KEGG) database ^2^ (Kanehisa et al., 2019), and for predominant protein-protein interaction networks according to the STRING database (Szklarczyk et al., 2017). These resources are publicly accessible and implemented in STRING ^3^. For our analyses, we used STRING at the level of highest confidence (0.900), with protein-protein interactions restricted to experimental evidence, and without addition of interactors at the first or second shell. Biological processes or pathways were considered significantly enriched after correction for multiple testing if the false discovery rate (FDR) was FDR < 0.05. Enrichment of GO processes was additionally validated in an independent analysis using the newest version 14.0 of the PANTHER (Protein ANalysis THrough Evolutionary Relationship) resource (Mi et al., 2019).

Additional analysis of networks and pathways were conducted using the commercial Ingenuity Pathway Analysis (IPA) software tool (Qiagen, Hilden, Germany). The standard setup for network analysis provided by the IPA core analysis was employed. The “molecules per network” parameter was set at 70, because of the large amount of input genes. All genes carrying single and multiple methylated CpG dinucleotides were used for the IPA analysis.

### Statistical Analysis

Statistical analysis of the Illumina methylation bead array data was performed separately for cell culture passage 3 and 5 with the R software (R version 3.5.0; R Core Team) following the analysis pipeline provided by the ChAMP package (Morris et al., 2014); probes were filtered for detection *p*-values above 0.01 removing 3334 probes. Overall, no completely failed measurements were identified. Using general EPIC SNP list for filtering probes with SNPs as identified by Zhou et al. (2017), 79762 probes were excluded from further analysis. Furthermore, 49 samples associated with more than one location as identified by Nordlund et al. (2013) were removed. Finally, the samples located on the X and Y chromosome were excluded from further analysis. Type-2 probe correction was carried out with the BMIQ method (Teschendorff et al., 2013). There were no significant batch effects to correct. After purification of the data the genomic methylation pattern of fetal ECFC from preeclamptic and uncomplicated control pregnancies, at low and advanced cell culture passages, were compared. For the identification of Differentially Methylated Regions (DMR) Probe Lasso method was used (Butcher and Beck, 2015).

## Results

### Differentially Methylated CpG Regions in Cord Blood ECFC Associated With Maternal Preeclampsia

We first compared the methylation patterns of cord blood derived ECFC associated with preeclampsia and normal pregnancy controls by means of the Infinium EPIC BeadChip microarray. This methylome analysis identified a number of differently regulated CpG sites, the majority of which were hypomethylated in fetal ECFCs from preeclamptic compared to control pregnancies.

**FIGURE 1:**
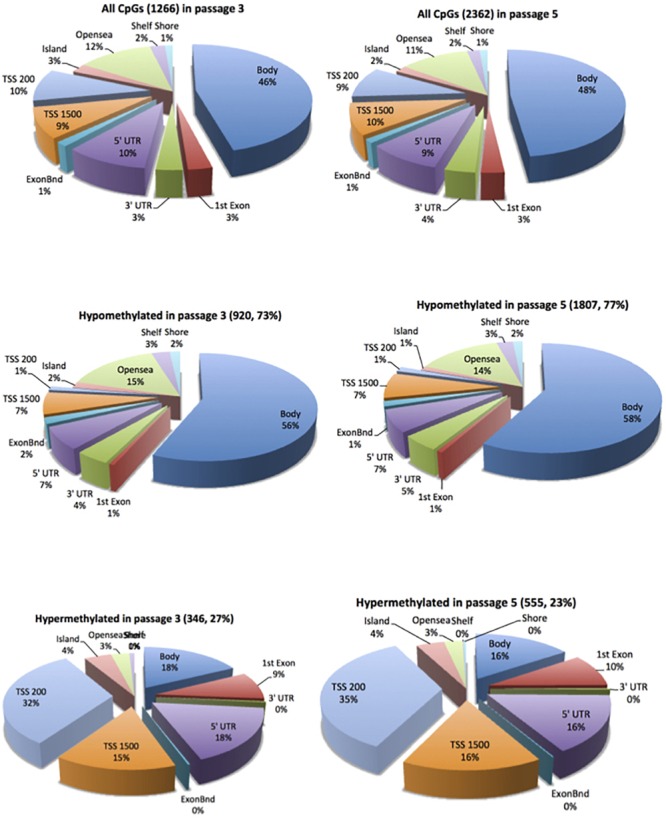
Distribution of the differentially methylated CpG dinucleotides in ECFC from pregnancies complicated by preeclampsia vs. controls. Top panel all differentially methylated CpG dinucleotides; mid panel hypomethylated CpG dinucleotides; bottom panel hypermethylated CpG dinucleotides. The exploded portion of the pie charts report the classification of those differentially methylated CpG dinucleotides that map in or near know genes. The other portion of the pie chart reports the main characteristics of the differentially methylated CpG dinucleotides that do not map in or near known genes.

**FIGURE 2:**
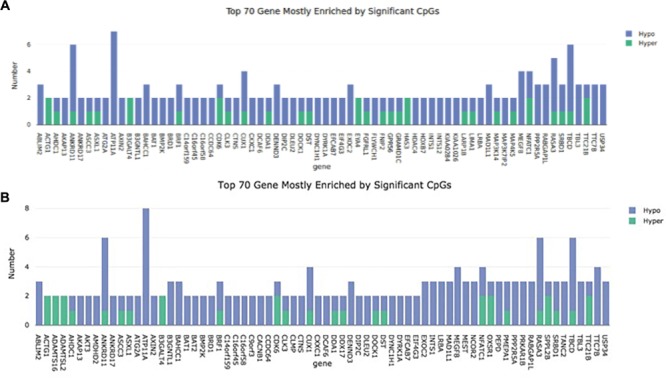
Representation of the 70 mostly methylated genes. The number of methylation sites is plotted on the *Y* axis. The gene name can be found on the *X* axis. **(A)** Cell passage 3; **(B)**: Cell passage 5.

In cell passage 3 a total of 346 hypermethylated and 920 hypomethylated CpG dinucleotides were identified in the preeclamptic group compared to the control group. Thirty out of 346 hypermethylated and 204 out of 920 hypomethylated CpGs mapped to regions not associated with any known gene. We identified 316 out of 346 hypermethylated and 716 out of 920 hypomethylated CpG dinucleotides that mapped in or near 954 known genes (Figure 1). Of these 954 genes, 891 were affected by a single altered CpG site. Sixty-three genes were altered by 2 or more sites. Figure 2A shows the 70 most differently methylated genes in the preeclamptic group compared to the control group.

In cell culture passage 5 we found 555 hypermethylated and 1807 hypomethylated CpG dinucleotides in the preeclamptic group compared to the control group. Here, 43 out of 555 hypermethylated and 351 out of 1807 hypomethylated CpGs mapped to regions not associated with any known gene. We identified 512 out of 555 hypermethylated and 1456 out of 1807 hypomethylated GpG mapped in or near 1719 known genes (Figure 1). Analogous to cell culture passage 3, 1530 genes were affected by a single altered CpG site and 199 genes were altered by 2 or more sites. Figure 2B shows the 70 most differently methylated genes in the preeclamptic group compared to the control group.

### Pathway Analysis Comparing Preeclampsia Derived ECFCs and Controls

**FIGURE 3:**
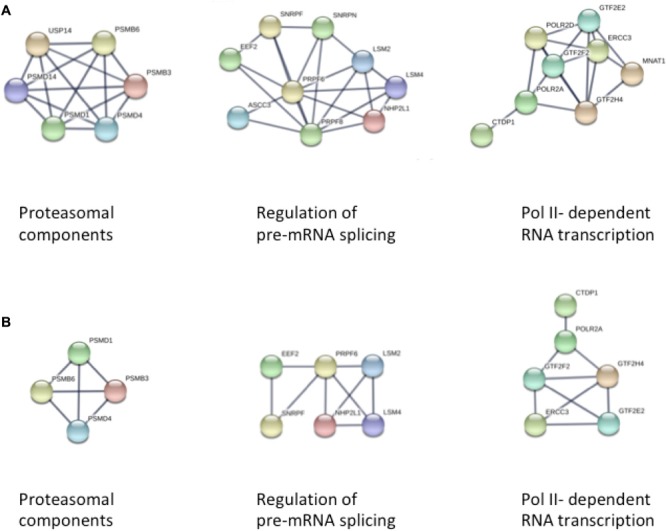
STRING analysis of protein-protein interaction networks in panel **(A)** Passage 5, and panel **(B)** Passage 3 ECFCs, respectively. In both analyses, three clusters of interacting proteins were identified that were related to proteasomal function, pre-mRNA splicing, and DNA-dependent RNA transcription (from left to right).

We first used STRING to explore the larger set of differentially methylated genes in passage 5 for an enrichment of biological processes according to the GO database. Subsequently we investigated for an enrichment of biological pathways as defined in the KEGG database, and for predominant protein-protein interaction networks. Among 1625 proteins differentially expressed in passage 5, we found a significant enrichment for 382 GO biological processes (Supplementary Table S1). There was a marked enrichment for proteins involved in primary metabolic processes (GO: 0044238, FDR 6.75 × 10^-16^), more specifically in the positive regulation of RNA metabolic processes (GO: 0051254, FDR 5.94 × 10^-10^), in cellular protein modification processes (GO: 0006464, FDR 5.29 × 10^-10^), and in the positive regulation of transcription (GO: 0045893, FDR 1.01 × 10^-9^). Consistent with this, the protein-protein interaction clusters with most nodes were observed for proteasomal function, RNA transcription and pre-mRNA splicing (Figure 3A). Enriched biological processes also included cell cycle (GO: 0007049, FDR 2.24 × 10^-9^), cellular nitrogen compound metabolism (GO: 0034641, FDR 1.04 × 10^-6^), adherens junction assembly (GO: 0034333, FDR 9.27 × 10^-5^), chromatin modification (GO: 0016568, FDR 1.27 × 10^-4^), cellular response to growth factor stimulus (GO: 0071363, FDR 6.23 × 10^-4^) and tube morphogenesis (GO: 0035239, 7.77 × 10^-4^). These observations point to a broad spectrum of enriched cellular developmental processes. KEGG pathway analyses indicated enrichment for 33 biological pathways, with the most significantly enriched pathways related to cell-cell interaction (5205, 4510, 4520, FDR 0.002–0.005), Wnt signaling (4310, FDR 0.003) and mTOR signaling (4150, FDR 0.004) (Supplementary Table S2). Estrogen signaling (FDR 0.01) and oxytocin signaling (FDR 0.03) were also among the enriched pathways.

The smaller subset of 899 proteins differentially expressed in passage 3 recapitulated to a large extent the findings from passage 5, with most pronounced enrichments for proteins involved in primary metabolic processes (GO: 0044238, FDR 8.4 × 10^-9^), cell surface receptor signaling (GO: 0007166, FDR 3.21 × 10^-7^), protein phosphorylation (GO: 0006468, FDR 5.77 × 10^-7^) and cell cycle (GO: 0005049, FDR 5.77 × 10^-7^) (Supplementary Tables S3, S4). Again, the three most extended protein interaction networks were found for proteins involved in proteasomal, transcriptional and pre-mRNA splicing processes, although the clusters were smaller in concordance with the smaller number of differentially methylated loci in passage 3 (Figure 3B).

We also performed IPA to evaluate and classify genes influenced by significantly altered CpG regions. IPA recognized 552 out of 954 injected genes in cell culture passage 3 and 1038 out of 1719 injected genes in cell culture passage 5. For the creation of a gene network, only the known genetic associations and linkages for mammalian endothelial cells from the IPA database were used. Both the significantly altered CpGs from preeclampsia compared to the control group from cell culture passage 3 and passage 5 were read and evaluated in parallel manner.

**FIGURE 4:**
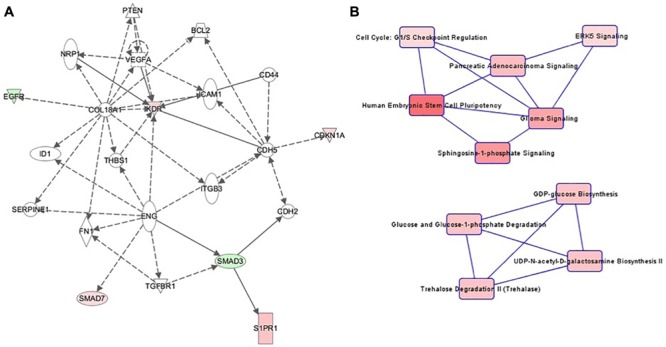
**(A)** Ingenuity pathway analysis (IPA) for the genes included in network of cell culture passage 3. Gene symbol shading: green, hypomethylated; red, hypermethylated; white, no differential methylation. For hyper- and hypomethylated genes, red/green gradient relates to the methylation level. **(B)** IPA canonical pathway analysis for the genes included in network for cell culture passage 3. Canonical pathway red symbol gradient relates to the *p*-value for the likelihood of the association between the differentially methylated genes in our experiment and the pathway. The smaller the *p*-value, the darker the red shading and the stronger the association.

The highest-ranking network for cell culture passage 3 was the “Cardiovascular System Development and Function, Organizational Development, Cellular Development” network (Figure 4A). This network comprises 22 genes, of which 6 in the preeclamptic samples are methylated significantly differently in comparison to the control samples (*CDKN1A: hypermethylated at 1 position, EGFR: hypomethylated at 1 position, KRD: hypermethylated at 1 position, S1PR1: hypermethlyated at 1 position, SMAD3: hypomethylated at 1 position, SMAD7: hypermethylated at 1 position*). The most important canonical pathways of the altered genes are summarized in Table 2.

**Table 2:** Summary of the ingenuity pathway analysis (IPA) network and pathway analysis.

Cell culture passage	Main Network	Main characteristics	Main genes	Other genes	Main pathways	Top upstream regulator
3	Cardiovascular System Development and Function, Organismal Development, Cellular Development	Gene (%): 6 (27) Hypo (%): 2 (9) Hyper (%): 4 (18)	*SMAD2* *SMAD3* *SMAD6* *SMAD7* *S1PR1* *S1PR4* *KDR* *MEF2* *PDGF* *p21* *EGFR* GCK	*ARHGAP22* *RASSF5* *SEMA3C* *PTPRZ1* *SLC29A1* *PDGFD* *CD2AP* *OSMR* *SCARB1* *PLXNB2* *LTBP1* *PLD1* *FERMT3* *CLK3* *ADAM10* *BCAR3* *EDG*	Human Embryonic Stem Cell Pluripotency Cell Cycle: G1/S Checkpoint Regulation Sphingosine-1-phosphate Signaling ERK5 Signaling Glucose and Glucose-1phosphate Degradation GDP-glucose Biosynthesis	CXXC5 ENG
5	Cardiovascular System Development and Function, Organismal Development, Cellular Development	Gene (%): 13 (30) Hypo (%): 6 (14) Hyper (%): 7 (16)	*SMAD2* *SMAD3* *SMAD6* *SMAD7* *S1PR1* *S1PR4* *KDR* *MEF2* *CDKN1A* *JAG2* *EGFR*	*SDC4* *RASSF5* *PTH1R* *PTPRZ1* *CBP* *CREB* *PLD* *ITGB1* *SEMA3C* *ARHGAP22* *PLPP3* *FLNB* *CD2AP* *FLNB* *OSMR* *ALCAM* *SCARB1* *PLXNB2* *LTBP1* *IL15* *ZDHHC7*	Human Embryonic Stem Cell Pluripotency Cell Cycle: G1/S Checkpoint Regulation Sphingosine-1-phosphate Signaling ERK5 Signaling Phospholipase C Signaling Protein Kinase A Signaling Regulation of the Epithelial-Mesenchymal Transition Pathway Glucose and Glucose-1phosphate Degradation GDP-glucose Biosynthesis ERK5 Signaling	BMPR2 ERG CXXC5
Genes changed in passage 5, but not in passage 3	Cellular Development, Cellular Function and Maintenance, Cellular Growth and Proliferation		*CREB* *GPCR* *CBP* *SMAD4*	*SDC4* *PTH1R* *ITGB1* *CLK3* *SCUBE2* *SLC2A6* *FLNB* *ALCAM* *IL15* *ZDHHC7* *LTBP2* *JAG2* *PEG10* *ADGRG1* *COL4A1* *PLXNB1*	G-alpha Signaling Rho GDI Signaling Granzyme A Signaling	ERG

*Genes (%) = number and percent of the genes of each network belonging to the list of genes carrying differentially methylated CpG dinucleotides from this study; Hypo (%) = number and percentage of genes carrying hypomethylated CpG dinucleotides (percentage calculated over the per-network total number of genes carrying differentially methylated CpG dinucleotides from this study); Hyper (%) = number and percentage of genes carrying hypermethylated CpG dinucleotides (percentage calculated over the per-network total number of genes carrying differentially methylated CpG dinucleotides from this study). Genes are reported in capital letters italicized; domains are reported as per the IPA notation.*

The canonical pathway analysis of the IPA data yielded 2 main webs (Figure 4B). The most influential and central pathway of the first web was “Human Embryonic Stem Cell Pluripotency,” a pathway with an important role in cell growth, proliferation and development. In the preeclamptic group compared to controls, *SMAD3* was significantly hypomethylated and *SMAD7* hypermethylated. SMAD activity is also part of the second pathway called “Cell Cycle: G1/S Checkpoint Regulation.” For instance, preeclampsia was associated with a hypermethylation of *CDKN1A*, encoding the cyclin dependent kinase inhibitor p21^Waf1/Cip1^. Further pathways in web 1 included “Sphingosine-1-phosphate Signaling” and “ERK5 Signaling.”

The second web was dominated by glucose metabolism, especially glucose and glucose-1-phosphate degradation and GDP-glucose biosynthesis.

**FIGURE 5:**
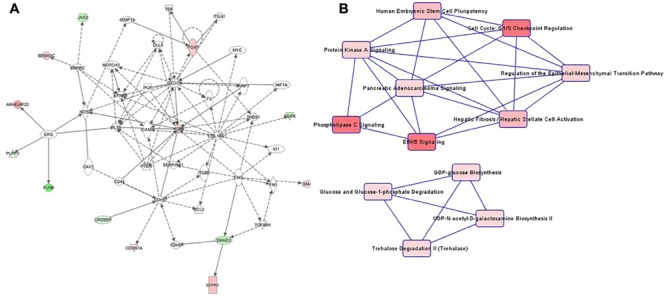
**(A)** Ingenuity pathway analysis (IPA) for the genes included in network of cell culture passage 5. Gene symbol shading: green, hypomethylated; red, hypermethylated; white, no differential methylation. For hyper- and hypomethylated genes, red/green gradient relates to the methylation level. **(B)** IPA canonical pathway analysis for the genes included in network for cell culture passage 5. Canonical pathway red symbol gradient relates to the *p*-value for the likelihood of the association between the differentially methylated genes in our experiment and the pathway. The smaller the *p*-value, the darker the red shading and the stronger the association.

Patterns in cell culture passage 5 were similar to cell culture passage 3. Again, the highest-ranking network was “Cardiovascular System Development and Function, Organizational Development, Cellular Development” (Figure 5A), comprising 44 genes, of which seven were hypermethylated and six genes were hypomethylated. The canonical pathway analysis yielded results similar to cell culture passage 3 (Figure 5B). However, pathway web 1 was extended by the pathway “protein kinase A signaling” and “phospholipase C signaling.” An additional significant epigenetic change detected in cell culture passage 5 but not yet in cell culture passage 3 was the pathway called “regulation of the epithelial-mesenchymal transition pathway.” In the preeclamptic setting PDGF was hypermethylated and EGFR hypomethylated, changes with potential to trigger a mesenchymal phenotype.

### Cell Passage Specific Methylation Analysis

**FIGURE 6:**
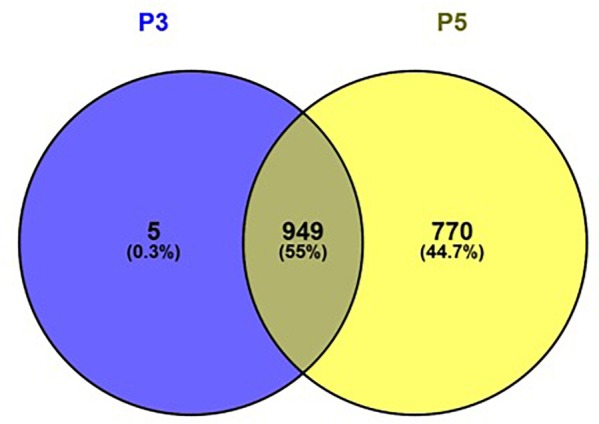
Venn diagram showing the overlap between differentially methylated coding genes in passage 3 cells (left) and passage 5 cells (right). Figure generated using Venny 2.1.0 (http://bioinfogp.cnb.csic.es/tools/venny).

Among all samples irrespective of pregnancy outcome group, a total of 1096 CpG regions were significantly altered in cell culture passage 5 compared to cell culture passage 3. Of these, 770 were gene-associated. Interestingly, only five gene-associated altered CpG regions, which were detectable in cell culture passage 3, were no longer detectable in passage 5. All other CpGs altered in passage 3 were also significantly altered in passage 5. The overlap between differentially methylated coding genes in passage 3 cells and passage 5 cells and the ratio of the coding genes in passage 3, passage 5 and passages 3 + 5 in percent is shown in Figure 6. By IPA pathway analysis, those genes impacted in passage 5 but not yet in passage 3 were assignable to the domain of “Cellular Development, Cellular Function” and “Maintenance, Cellular Growth and Proliferation.” The canonical pathway analysis revealed “G-alpha signaling” (which regulates signal transduction and thus initiates changes in cell behavior) “Rho-GDI signaling” (important for cell differentiation, proliferation and apoptosis), and “Sumoylation pathway” (which plays a role in cell cycle control, trafficking and apoptosis). These pathways can be broadly categorized as intracellular and second messenger signaling, and cellular growth and transcriptional regulation.

## Discussion

The molecular and cellular dysfunctions associated with preeclampsia are still largely unknown. Here we compared the global DNA methylation profiles of fetal ECFCs from uncomplicated pregnancies and pregnancies complicated by preeclampsia. We identified 954 genes in passage 3 and 1719 genes in passage 5 with significantly differentially methylated CpG sites between the two groups. Almost all changes between the group with preeclampsia and the control group detected in cell culture passage 3 were also confirmed in cell culture passage 5 (1260 out of 1266), indicating that the methylation differences in passage 3 are stably propagated and methylation dysregulation accumulates at later passages. Several studies showed that higher cell passage numbers lead to more inconsistent production of certain biomarkers and methylation levels at least in cancer cells and endocrine cells (Kwist et al., 2016; Hamadneh et al., 2018). An analysis of the effect of cell culture passage revealed very similar results in passage 3 and passage 5 in terms of the profile of the gene networks and the pathways associated with preeclampsia.

Hypermethylation predominantly results in a decrease in gene expression whereas hypomethylation tends to be gene activating (Schubeler, 2015). Interestingly, much of the epigenetic changes in our study involved hypomethlylation. This result is consistent with studies of methylation in placentas from women with preeclampsia (Yuen et al., 2010; Blair et al., 2013; Martin et al., 2015). These reports indicated a general loss of CpG methylation in preeclampsia placentas, such that the profile was significantly different from normotensive, uncomplicated pregnancies. We found a substantial number of the methylation differences to reside in non-coding regions of the genome. This is unlikely to be unique to our model system given that changes in non-coding regions have been shown to be associated with the overexpression of long non-coding RNAs (lncRNAs) transcripts. These RNAs, characterized as a novel class of non-coding RNAs are linked to human disease and exerting specific functions (Mattick, 2004; Wu et al., 2013). In addition, lncRNAs may regulate gene function by influencing splicing, transport, translation or degradation of their corresponding mRNA (Yu et al., 2008). Accumulated evidence suggests that many lncRNAs are expressed abnormally in placents from preeclamptic pregnancies and that they may play a role in functional development of preeclampsia (Chen et al., 2015; Song et al., 2017; Xu et al., 2017).

Our results for both GO biological processes and protein-protein interaction networks indicated that ECFCs from preeclamptic pregnancies are differentially methylated in regions corresponding to a broad range of processes regulating cell metabolism, transcription and cell cycle, with a likely role for protein networks involved in proteasomal, transcriptional and pre-mRNA splicing processes. KEGG pathway analyses pointed to differential regulation of cell-cell interaction, Wnt signaling and mTOR signaling. In regard to preeclampsia, these pathways might be expected to negatively impact the capacity for endothelial development/repair and trophoblast invasion. Our results from IPA, restricted to interactions in mammalian endothelial cells, revealed “Cardiovascular System Development and Function, Organizational Development, Cellular Development” as the highest ranking network.

The methylation pattern of fetal ECFC reveals a unique epigenetic signature which may represent a close link to cardiovascular system development and function and contribute to the cardiovascular risk of offspring born of preeclamptic pregnancies. As this is a study on fetal ECFC we can imagine that epigenetic modules may act as a molecular readout of cumulative cardiovascular risk factor exposure, with implications for the improvement of clinical risk prediction in the future. However, this has never been proven and follow up studies on these children from preeclamptic pregnancies in a larger cohort are clearly necessary. Comprehensive reviews and studies also suggest that changes in DNA methylation states contribute to the regulation of biological processes underlying cardiovascular disease, such as atherosclerosis, hypertension, and inflammation (Baccarelli et al., 2010) and several studies in adults show epigenetic associations with incident cardiovascular disease that reveal disease mechanisms (Friso et al., 2008; Turunen et al., 2009). Also, integrated models using genetic, epigenetic and phenotype data are able to detect symptomatic cardiovascular disease (Dogan et al., 2018). However, we are unaware of any study using methylation data that predicted cardiovascular disease before symptomatic disease occurred.

Studies on the methylation of human gestational tissue, e.g., placental tissue or cells derived from umbilical cord blood are only at the beginning. Therefore, this project is novel in both the cells studied and the implications of fetal progenitor cells for pathogenesis of preeclampsia and its long-term consequences for the offspring. An epigenetically modified endothelial precursor could affect both normal feto-placental vascular development and repair capacity and might influence the subsequent health of the offspring. Although implications for maternal pathogenesis of preeclampsia can not be inferred by our data, there is evidence that fetal ECFCs have a role in normal placental vasculogenesis and further that these cells traverse the placenta and home to sites of vessel formation in the maternal uterine microvasculature (Sipos et al., 2013).

Our data, while in need of further exploration, suggest involvement of certain pathways in the epigenetic imprinting of preeclampsia in utero. The main canonical pathway implicated deals with embryonic stem cell pluripotency and is regulated by extrinsic and intrinsic factors. Here the regulation of TGF-beta signaling via SMAD proteins is in center. The hypo- and hypermethylation of SMAD coding genes changes in cell differentiation associated with preeclampsia Linked through SMAD activity a second important pathway for cell cycle checkpoint regulation was observed. The key components involved are the cell cycle kinases and the transcription complex. The bioactive sphingosine-1-phosphate has been linked to a wide spectrum of biological processes, including cell growth, survival and motility and forms the bridge to pathway 3. The hypermethylation of this central gene in this pathway can possibly lead to a reduction of cell survival and migration capacity via the described pathway (Dobierzewska et al., 2016). The ligand sphingosine-1-phosphate also seems to be influenced indirectly via the methylation of platelet derived growth factor (PDGF) and a lack of stimulation of sphingosine-kinase-1 in the preeclamptic setting (Soliven et al., 2003). Another affected pathway was ERK5 signaling. Typically, it is activated by external stimuli such as serum, growth factors, and cellular stressors including hypoxia and altered osmotic pressure (Busch et al., 2018). Simplified, activation results in increased gene expression and cell survival. In the preeclampsia group, two central genes of this pathway, namely the EGF-receptor and the myocyte enhancer factor 2 (MEF2), were hypomethylated, suggesting that a change in gene products and thus activation of the pathway is possible under the stressor preeclampsia. Further pathways were found including glucose and glucose-1-phosphate degradation and GDP-glucose biosynthesis, whose activation may occur with preeclampsia.

Given the potential utility of ECFCs in cell-based therapies, a subanalysis focused on the comparison of two different cell culture passages to assess whether the cell culture passage affects the methylation pattern. Cell culture passage 3 corresponds to the earliest time at which a sufficient number of cells can be harvested after the initial isolation. Passage 5 was chosen as a comparison group, as ECFC at this cell age are commonly used for functional studies. Although the results in cell culture passage 3 and 5 were similar in terms of both the patterns of the gene networks and the pathways, some differences were nevertheless detected. The differentially methylated genes by cell culture passages could be assigned to cellular development, function and maintenance. The canonical analysis revealed pathway differences which regulate signal transduction, cell behavior and differentiation, proliferation and apoptosis. Based on these results, it can be interpreted that the culture of ECFC leads to a change in the methylation profile primarily of cell culture specific pathways. As almost all differences between control group and preeclampsia group detected in cell culture passage 3 were also confirmed in cell culture passage 5 (1260 out of 1266), the gene modification caused by preeclampsia appeared to be largely stable through higher cell culture passages.

IPA results suggest that there are similarities between epigenetic changes in preeclampsia and cardiovascular disease outside the setting of pregnancy. Cardiovascular diseases are complex multifactorial and polygenetic, with interaction of numerous genes, genetic variants, and environmental factors contributing to development (Vukasinovic et al., 2009). In attempts to demonstrate association between genetic variants and cardiovascular disease, researchers have run large genome wide association studies. In our study, we identified important epigenetic changes already described in the pathogenesis of preeclampsia, or whose activation or inactivation of their gene products fit to well-known or suspected pathways in preeclampsia (Ashur-Fabian et al., 2012; Armant et al., 2015; Almasry et al., 2016; Meyer et al., 2016). We also tabulated altered genes of known relevance to cardiovascular disease (Table 3). The principal genes in the analysis could plausibly play a role in preeclampsia pathogenesis and in later future cardiovascular diseases of the offspring.

**Table 3:** Overlapping findings between the existing cardiovascular literature and the work presented in this study.

Gene; full name	Description in NCBI gene data base	Known function in preeclampsia	Known function in cardiovascular disease	Status in preeclampsia-derived compared to normal pregnancy-derived ECFCs
*EGFR*; epidermal growth factor receptor	Cell surface protein that binds to epidermal growth factor. Binding to a ligand induces receptor dimerization and tyrosine autophosphorylation and leads to cell proliferation.	Disruption of the EGF signaling system seems to contribute to aberrant trophoblast development (Armant et al., 2015).	EGFR blockade reduces atherosclerosis development (Zeboudj et al., 2018).	Hypomethylated
*KDR*; kinase insert domain receptor	Type III receptor tyrosine kinase that functions as the main mediator of VEGF-induced endothelial proliferation, survival, migration, tubular morphogenesis and sprouting.	VEGFR-1 mediates trophoblast function and inhibits VEGF-induced angiogenesis and endothelium-dependent vasodilation (Chung et al., 2004). Altered tissue expression of VEGF and its receptors are described in the umbilical cord vessels from preeclamptic pregnancies (Almasry et al., 2016).	VEGF and its receptors play a role in vascular aging, peripheral artery disease, coronary vascular disease and atherosclerotic plaque growth (Clegg et al., 2017; Yang et al., 2017; Kurotsu et al., 2018).	Hypermethylated
*PDGF*; platelet derived growth factor	Mitogenic factor for cells of mesenchymal origin.	PDGF is associated with the pathology of decidual blood vessel. Elevated serum PDGF levels and PDGF-B mRNA expression in the decidual blood vessel may play an important role in the pathogenesis of preeclampsia (Meng et al., 2007).	Altered expression of the receptors and the ligands have been found in various cardiovascular diseases and PDGF-C and PDGF-D signaling has been implicated in fibrosis, neovascularization, atherosclerosis and restenosis (Folestad et al., 2018; Lee and Li, 2018).	Hypermethylated
*SMAD2*; SMAD family member 2	Mediator of transforming growth factor (TGF)-beta, regulating cell proliferation, apoptosis, and differentiation.	Reduced SMAD2 phosphorylation in a preeclampsia animal model and placental tissues. Endothelial cells may be protected from hypoxia injury through SMAD2 axis (Feng et al., 2017).	Regulation of artery size, cardiomyogenesis and vascular smooth muscle cell migration in atherosclerosis (Perino et al., 2017; Poduri et al., 2017; Pan et al., 2018).	Hypomethylated
*S1PR1*/4; sphingosine-1-phosphate receptor 1 and 4	Involved in the processes that regulate the differentiation of endothelial cells. Activation of this receptor induces cell-cell adhesion.	SPHK pathway may play a role in the early placentation process and may be involved in the pathogenesis of preeclampsia (Dobierzewska et al., 2016).	S1PR1 signaling regulates blood flow and pressure, induces cardiac hypertrophy and fibrosis and building of atherosclerotic plaques (Cantalupo et al., 2017; Ohkura et al., 2017; Liu et al., 2018).	Hypermethylated
*CDKN1A*; cyclin dependent kinase inhibitor 1A	Regulator of cell cycle progression at G1. This protein also plays a regulatory role in S phase DNA replication and DNA damage repair.	Involved in cell growth, G1 arrest and apoptosis in endothelium from preeclamptic pregnancies (Ashur-Fabian et al., 2012; Gao et al., 2016).	Implicated in many cardiovascular processes, such as cardiac hypertrophy, proliferation and migration of vascular smooth muscle cells or M myocardial fibrosis (Meyer et al., 2016; Pei et al., 2016; Tong et al., 2017).	Hypermethylated
*JAG2*; jagged 2	Activator of the Notch signaling pathway, an intercellular signaling mechanism essential for proper embryonic development.	Involved in the regulation of trophoblast fate decisions, vasculogenesis and feto-maternal trafficking (Herr et al., 2011).	Role in cardiac regeneration process, ventricular chamber development and cardiomyopathy (Samal et al., 2012; D’Amato et al., 2016).	Hypomethylated
*MEF2*; myocyte enhancer factor 2A	DNA-binding transcription factor that activates muscle-specific, growth factor-induced, and stress-induced genes.	Dysregulated expression may be associated with placenta-related pregnancy disorders (Li et al., 2018).	Key regulator of sprouting angiogenesis (Sacilotto et al., 2016). Besides it plays a key role in cardiomyocyte survival (Hashemi et al., 2015).	Hypomethylated

As a note of caution, the IPA results were obtained from an endothelium-restricted database of interactions, and thus the emergence of a cardiovascular pathway could be partially attributed to these presettings. However, our unrestricted STRING analyses also identified clear links to the cardiovascular system, such as the GO term “tube morphogenesis” that includes angiogenesis, or the Wnt signaling pathway known to regulate the mobilization and proliferation of cells in endothelium and epicardium in an ischemic heart (Oerlemans et al., 2010; Deb, 2014), thus adding weight to the possible overlap of predisposing factors between preeclampsia, a hypertensive disorder, and the future risk of cardiovascular disease.

The altered methylation profile of fetal ECFC may have been present prior to the onset of clinical manifestations of preeclampsia. If already present, they could contribute to risk of preeclampsia. However, the changes could also be caused by the preeclampsia milieu, i.e., by oxidative stress and DNA oxidation (Weitzman et al., 1994; Hitchler and Domann, 2007; Franco et al., 2008).

To the best of our knowledge, our study is the first to explore whether the human intrauterine environment in preeclampsia is associated with abnormal fetal ECFC epigenetic gene network programming. These data suggest that fetal ECFC methylation status differs between preeclampsia and uncomplicated pregnancy. Our pathway analysis of the genes impacted by the methylation regulation may provide insights into possible mechanisms of pathogenesis. Future studies are needed to clarify which factors impact specific methylation changes, which methylation changes are involved in various obstetric complications, and what bearing these changes might have on offspring health.

## Author Contributions

LB and FvV-H conceived and designed the experiments. LB and SvH performed the experiments. LB, TZ, TD, and HF analyzed the data. FvV-H, CH, and CvK contributed reagents, materials and analysis tools. LB, FvV-H, CH, and BS-H contributed to the writing of the manuscript.

## Conflict of Interest Statement

The authors declare that the research was conducted in the absence of any commercial or financial relationships that could be construed as a potential conflict of interest.

## Footnotes

1 www.geneontology.org/

2 www.genome.jp/kegg/

3 www.string-db.org/

## Supplementary Material

TABLE S1 List of Gene Ontology biological processes that were significantly enriched (FDR < 0.05) in passage 5 ECFC from preeclamptic patients versus passage 5 ECFC from healthy donors.

TABLE S2 List of Gene Ontology biological processes that were significantly enriched (FDR < 0.05) in passage 3 ECFC from preeclamptic patients versus passage 3 ECFC from healthy donors.

TABLE S3 List of KEGG pathways that were significantly enriched (FDR < 0.05) in passage 5 ECFC from preeclamptic patients versus passage 5 ECFC from healthy donors.

TABLE S4 List of KEGG pathways that were significantly enriched (FDR < 0.05) in passage 3 ECFC from preeclamptic patients versus passage 3 ECFC from healthy donors.

## References

[B1] ACOG Committee on Practice Bulletins–Obstetrics (2002). ACOG practice bulletin. Diagnosis and management of preeclampsia and eclampsia. Number 33, January 2002. *Obstetr. Gynecol.* 99 159–167. 10.1016/s0029-7844(01)01747-1 16175681

[B2] Almasry S. M. Elfayomy A. K. Hashem H. E. (2016). Ultrastructure and histomorphometric analysis of human umbilical cord vessels in preeclampsia: a potential role of VEGF, VEGFR-1 and VEGFR-2. *Rom. J. Morphol. Embryol.* 57(2 Suppl.) 681–689. 27833959

[B3] Armant D. R. Fritz R. Kilburn B. A. Kim Y. M. Nien J. K. Maihle N. J. (2015). Reduced expression of the epidermal growth factor signaling system in preeclampsia. *Placenta* 36 270–278. 10.1016/j.placenta.2014.12.006 25589361 PMC4331205

[B4] Ashur-Fabian O. Yerushalmi G. M. Mazaki-Tovi S. Steinberg D. M. Goldshtein I. Yackobovitch-Gavan M. (2012). Cell free expression of hif1alpha and p21 in maternal peripheral blood as a marker for preeclampsia and fetal growth restriction. *PLoS One* 7:e37273. 10.1371/journal.pone.0037273 22615960 PMC3353943

[B5] Baccarelli A. Rienstra M. Benjamin E. J. (2010). Cardiovascular epigenetics: basic concepts and results from animal and human studies. *Circ. Cardiovasc. Genet.* 3 567–573. 10.1161/CIRCGENETICS.110.958744 21156932 PMC3030456

[B6] Barker D. J. Winter P. D. Osmond C. Margetts B. Simmonds S. J. (1989). Weight in infancy and death from ischaemic heart disease. *Lancet* 2 577–580. 10.1016/S0140-6736(89)90710-1 2570282

[B7] Blair J. D. Yuen R. K. Lim B. K. McFadden D. E. von Dadelszen P. Robinson W. P. (2013). Widespread DNA hypomethylation at gene enhancer regions in placentas associated with early-onset pre-eclampsia. *Mol. Hum. Reprod.* 19 697–708. 10.1093/molehr/gat044 23770704 PMC3779005

[B8] Brodowski L. Burlakov J. Myerski A. C. von Kaisenberg C. S. Grundmann M. Hubel C. A. (2014). Vitamin D prevents endothelial progenitor cell dysfunction induced by sera from women with preeclampsia or conditioned media from hypoxic placenta. *PLoS One* 9:e98527. 10.1371/journal.pone.0098527 24887145 PMC4041729

[B9] Busch M. Wasmuth S. Spital G. Lommatzsch A. Pauleikhoff D. (2018). Activation of the ERK1/2-MAPK signaling pathway by complement serum in UV-POS-Pretreated ARPE-19 cells. *Ophthalmologica* 239 215–224. 10.1159/000486404 29486466

[B10] Butcher L. M. Beck S. (2015). Probe Lasso: a novel method to rope in differentially methylated regions with 450K DNA methylation data. *Methods* 72 21–28. 10.1016/j.ymeth.2014.10.036 25461817 PMC4304833

[B11] Cantalupo A. Gargiulo A. Dautaj E. Liu C. Zhang Y. Hla T. (2017). S1PR1 (Sphingosine-1-Phosphate Receptor 1) signaling regulates blood flow and pressure. *Hypertension* 70 426–434. 10.1161/HYPERTENSIONAHA.117.09088 28607130 PMC5531041

[B12] Chen H. Meng T. Liu X. Sun M. Tong C. Liu J. (2015). Long non-coding RNA MALAT-1 is downregulated in preeclampsia and regulates proliferation, apoptosis, migration and invasion of JEG-3 trophoblast cells. *Int. J. Clin. Exp. Pathol.* 8 12718–12727. 26722461 PMC4680406

[B13] Chung J. Y. Song Y. Wang Y. Magness R. R. Zheng J. (2004). Differential expression of vascular endothelial growth factor (VEGF), endocrine gland derived-VEGF, and VEGF receptors in human placentas from normal and preeclamptic pregnancies. *J. Clin. Endocrinol. Metab.* 89 2484–2490. 10.1210/jc.2003-031580 15126581 PMC3282114

[B14] Clegg L. E. Ganta V. C. Annex B. H. Mac Gabhann F. (2017). Systems pharmacology of VEGF165b in peripheral artery disease. *CPT Pharmacometrics Syst. Pharmacol.* 6 833–844. 10.1002/psp4.12261 29193887 PMC5744173

[B15] Cockell A. P. Learmont J. G. Smarason A. K. Redman C. W. Sargent I. L. Poston L. (1997). Human placental syncytiotrophoblast microvillous membranes impair maternal vascular endothelial function. *Br. J. Obstet. Gynaecol.* 104 235–240. 10.1111/j.1471-0528.1997.tb11052.x 9070146

[B16] D’Amato G. Luxan G. de la Pompa J. L. (2016). Notch signalling in ventricular chamber development and cardiomyopathy. *FEBS J.* 283 4223–4237. 10.1111/febs.13773 27260948

[B17] Davis E. F. Lazdam M. Lewandowski A. J. Worton S. A. Kelly B. Kenworthy Y. (2012). Cardiovascular risk factors in children and young adults born to preeclamptic pregnancies: a systematic review. *Pediatrics* 129 e1552–e1561. 10.1542/peds.2011-3093 22614768

[B18] Deb A. (2014). Cell-cell interaction in the heart via Wnt/beta-catenin pathway after cardiac injury. *Cardiovasc. Res.* 102 214–223. 10.1093/cvr/cvu054 24591151 PMC3989450

[B19] Dignat-George F. Sampol J. Lip G. Blann A. D. (2003). Circulating endothelial cells: realities and promises in vascular disorders. *Pathophysiol. Haemost. Thromb.* 33 495–499. 10.1159/000083851 15692266

[B20] Dobierzewska A. Palominos M. Sanchez M. Dyhr M. Helgert K. Venegas-Araneda P. (2016). Impairment of angiogenic sphingosine kinase-1/Sphingosine-1-phosphate receptors pathway in preeclampsia. *PLoS One* 11:e0157221. 10.1371/journal.pone.0157221 27284992 PMC4902228

[B21] Dogan M. V. Grumbach I. M. Michaelson J. J. Philibert R. A. (2018). Integrated genetic and epigenetic prediction of coronary heart disease in the framingham heart study. *PLoS One* 13:e0190549. 10.1371/journal.pone.0190549 29293675 PMC5749823

[B22] Feng Y. Wang N. Xu J. Zou J. Liang X. Liu H. (2017). Alpha-1-antitrypsin functions as a protective factor in preeclampsia through activating Smad2 and inhibitor of DNA binding 4. *Oncotarget* 8 113002–113012. 10.18632/oncotarget.22949 29348884 PMC5762569

[B23] Folestad E. Kunath A. Wagsater D. (2018). PDGF-C and PDGF-D signaling in vascular diseases and animal models. *Mol. Aspects Med.* 62 1–11. 10.1016/j.mam.2018.01.005 29410092

[B24] Franco R. Schoneveld O. Georgakilas A. G. Panayiotidis M. I. (2008). Oxidative stress, DNA methylation and carcinogenesis. *Cancer Lett.* 266 6–11. 10.1016/j.canlet.2008.02.026 18372104

[B25] Friso S. Pizzolo F. Choi S. W. Guarini P. Castagna A. Ravagnani V. (2008). Epigenetic control of 11 beta-hydroxysteroid dehydrogenase 2 gene promoter is related to human hypertension. *Atherosclerosis* 199 323–327. 10.1016/j.atherosclerosis.2007.11.029 18178212

[B26] Gao Q. Zhu X. Chen J. Mao C. Zhang L. Xu Z. (2016). Upregulation of P53 promoted G1 arrest and apoptosis in human umbilical cord vein endothelial cells from preeclampsia. *J. Hypertens.* 34 1380–1388. 10.1097/HJH.0000000000000944 27115339 PMC5649442

[B27] Gene Ontology Consortium (2015). Gene ontology consortium: going forward. *Nucleic Acids Res.* 43 D1049–D1056. 10.1093/nar/gku1179 25428369 PMC4383973

[B28] Grundmann M. Haidar M. Placzko S. Niendorf R. Darashchonak N. Hubel C. A. (2012). Vitamin D improves the angiogenic properties of endothelial progenitor cells. *Am. J. Physiol. Cell Physiol.* 303 C954–C962. 10.1152/ajpcell.00030.2012 22932684 PMC3492823

[B29] Hamadneh L. Al-Majawleh M. Jarrar Y. Shraim S. Hasan M. Abu-Irmaileh B. (2018). Culturing conditions highly affect DNA methylation and gene expression levels in MCF7 breast cancer cell line. *In Vitro Cell. Dev. Biol. Anim.* 54 331–334. 10.1007/s11626-018-0245-7 29633080

[B30] Hashemi S. Wales S. Miyake T. McDermott J. C. (2015). Heart disease: recruitment of MEF2 activity by beta-blockers wards off cardiomyocyte death. *Cell Death Dis.* 6:e1916. 10.1038/cddis.2015.293 26469965 PMC4632314

[B31] Herr F. Schreiner I. Baal N. Pfarrer C. Zygmunt M. (2011). Expression patterns of Notch receptors and their ligands jagged and delta in human placenta. *Placenta* 32 554–563. 10.1016/j.placenta.2011.04.018 21726900

[B32] Hitchler M. J. Domann F. E. (2007). An epigenetic perspective on the free radical theory of development. *Free Radic. Biol. Med.* 43 1023–1036. 10.1016/j.freeradbiomed.2007.06.027 17761298 PMC2981179

[B33] Ingram D. A. Mead L. E. Tanaka H. Meade V. Fenoglio A. Mortell K. (2004). Identification of a novel hierarchy of endothelial progenitor cells using human peripheral and umbilical cord blood. *Blood* 104 2752–2760. 10.1182/blood-2004-04-1396 15226175

[B34] Ingram E. R. Robertson I. K. Ogden K. J. Dennis A. E. Campbell J. E. Corbould A. M. (2017). Utility of antenatal clinical factors for prediction of postpartum outcomes in women with gestational diabetes mellitus (GDM). *Austr. N. Zeal. J. Obstetr. Gynaecol.* 57 272–279. 10.1111/ajo.12514 27549600

[B35] Jayet P. Y. Rimoldi S. F. Stuber T. Salmon C. S. Hutter D. Rexhaj E. (2010). Pulmonary and systemic vascular dysfunction in young offspring of mothers with preeclampsia. *Circulation* 122 488–494. 10.1161/CIRCULATIONAHA.110.941203 20644018

[B36] Kanehisa M. Sato Y. Furumichi M. Morishima K. Tanabe M. (2019). New approach for understanding genome variations in KEGG. *Nucleic Acids Res.* 47 D590–D595. 10.1093/nar/gky962 30321428 PMC6324070

[B37] Kurotsu S. Osakabe R. Isomi M. Tamura F. Sadahiro T. Muraoka N. (2018). Distinct expression patterns of Flk1 and Flt1 in the coronary vascular system during development and after myocardial infarction. *Biochem. Biophys. Res. Commun.* 495 884–891. 10.1016/j.bbrc.2017.11.094 29158084

[B38] Kwist K. Bridges W. C. Burg K. J. (2016). The effect of cell passage number on osteogenic and adipogenic characteristics of D1 cells. *Cytotechnology* 68 1661–1667. 10.1007/s10616-015-9883-8 26208915 PMC4960171

[B39] Lee C. Li X. (2018). Platelet-derived growth factor-C and -D in the cardiovascular system and diseases. *Mol. Aspects Med.* 62 12–21. 10.1016/j.mam.2017.09.005 28965749

[B40] Li L. Rubin L. P. Gong X. (2018). MEF2 transcription factors in human placenta and involvement in cytotrophoblast invasion and differentiation. *Physiol. Genom.* 50 10–19. 10.1152/physiolgenomics.00076.2017 29127222 PMC5866412

[B41] Lin C. Rajakumar A. Plymire D. A. Verma V. Markovic N. Hubel C. A. (2009). Maternal endothelial progenitor colony-forming units with macrophage characteristics are reduced in preeclampsia. *Am. J. Hypertens.* 22 1014–1019. 10.1038/ajh.2009.101 19498340 PMC2830891

[B42] Liu H. Jin H. Han J. Yue X. Yang H. Zayed M. A. (2018). Upregulated sphingosine 1-Phosphate Receptor 1 expression in human and murine atherosclerotic plaques. *Mol. Imaging Biol.* 20 448–456. 10.1007/s11307-017-1141-3 29134505 PMC5975257

[B43] Luppi P. Powers R. W. Verma V. Edmunds L. Plymire D. Hubel C. A. (2010). Maternal circulating CD34+VEGFR-2+ and CD133+VEGFR-2+ progenitor cells increase during normal pregnancy but are reduced in women with preeclampsia. *Reprod. Sci.* 17 643–652. 10.1177/1933719110366164 20360595 PMC2893245

[B44] Martin E. Ray P. D. Smeester L. Grace M. R. Boggess K. Fry R. C. (2015). Epigenetics and preeclampsia: defining functional epimutations in the preeclamptic placenta related to the TGF-beta pathway. *PLoS One* 10:e0141294. 10.1371/journal.pone.0141294 26510177 PMC4624949

[B45] Mattick J. S. (2004). RNA regulation: a new genetics? *Nat. Rev. Genet.* 5 316–323. 10.1038/nrg1321 15131654

[B46] Maynard S. E. Min J. Y. Merchan J. Lim K. H. Li J. Mondal S. (2003). Excess placental soluble fms-like tyrosine kinase 1 (sFlt1) may contribute to endothelial dysfunction, hypertension, and proteinuria in preeclampsia. *J. Clin. Investig.* 111 649–658. 10.1172/JCI17189 12618519 PMC151901

[B47] Meng H. Zhu F. F. Wang C. H. Xiao G. X. (2007). Role of platelet-derived growth factor in the pathogenesis of preeclampsia. *Nan Fang Yi Ke Da Xue Xue Bao* 27 1274–1276. 17715047

[B48] Meyer K. Hodwin B. Ramanujam D. Engelhardt S. Sarikas A. (2016). Essential role for premature senescence of myofibroblasts in myocardial fibrosis. *J. Am. Coll. Cardiol.* 67 2018–2028. 10.1016/j.jacc.2016.02.047 27126529

[B49] Mi H. Muruganujan A. Ebert D. Huang X. Thomas P. D. (2019). PANTHER version 14: more genomes, a new PANTHER GO-slim and improvements in enrichment analysis tools. *Nucleic Acids Res.* 47 D419–D426. 10.1093/nar/gky1038 30407594 PMC6323939

[B50] Morris T. J. Butcher L. M. Feber A. Teschendorff A. E. Chakravarthy A. R. Wojdacz T. K. (2014). ChAMP: 450k chip analysis methylation pipeline. *Bioinformatics* 30 428–430. 10.1093/bioinformatics/btt684 24336642 PMC3904520

[B51] Murayama T. Asahara T. (2002). Bone marrow-derived endothelial progenitor cells for vascular regeneration. *Curr. Opin. Mol. Ther.* 4 395–402. 12222878

[B52] Nordlund J. Backlin C. L. Wahlberg P. Busche S. Berglund E. C. Eloranta M. L. (2013). Genome-wide signatures of differential DNA methylation in pediatric acute lymphoblastic leukemia. *Genome Biol.* 14:r105. 10.1186/gb-2013-14-9-r105 24063430 PMC4014804

[B53] Oerlemans M. I. Goumans M. J. van Middelaar B. Clevers H. Doevendans P. A. Sluijter J. P. (2010). Active Wnt signaling in response to cardiac injury. *Basic Res. Cardiol.* 105 631–641. 10.1007/s00395-010-0100-9 20373104 PMC2916122

[B54] Ohkura S. I. Usui S. Takashima S. I. Takuwa N. Yoshioka K. Okamoto Y. (2017). Augmented sphingosine 1 phosphate receptor-1 signaling in cardiac fibroblasts induces cardiac hypertrophy and fibrosis through angiotensin II and interleukin-6. *PLoS One* 12:e0182329. 10.1371/journal.pone.0182329 28771545 PMC5542600

[B55] Pan J. Lu L. Wang X. Liu D. Tian J. Liu H. (2018). AIM2 regulates vascular smooth muscle cell migration in atherosclerosis. *Biochem. Biophys. Res. Commun.* 497 401–409. 10.1016/j.bbrc.2018.02.094 29448104

[B56] Pei X. Li X. Chen H. Han Y. Fan Y. (2016). Thymoquinone inhibits angiotensin II-induced proliferation and migration of vascular smooth muscle cells through the AMPK/PPARgamma/PGC-1alpha pathway. *DNA Cell Biol.* 35 426–433. 10.1089/dna.2016.3262 27064837

[B57] Perino M. G. Yamanaka S. Riordon D. R. Tarasova Y. Boheler K. R. (2017). Ascorbic acid promotes cardiomyogenesis through SMAD1 signaling in differentiating mouse embryonic stem cells. *PLoS One* 12:e0188569. 10.1371/journal.pone.0188569 29232368 PMC5726630

[B58] Poduri A. Chang A. H. Raftrey B. Rhee S. Van M. Red-Horse K. (2017). Endothelial cells respond to the direction of mechanical stimuli through SMAD signaling to regulate coronary artery size. *Development* 144 3241–3252. 10.1242/dev.150904 28760815 PMC5612251

[B59] Redman C. W. Sargent I. L. (2005). Latest advances in understanding preeclampsia. *Science* 308 1592–1594. 10.1126/science.1111726 15947178

[B60] Roberts J. M. (2000). Preeclampsia: what we know and what we do not know. *Semin. Perinatol.* 24 24–28. 10.1016/S0146-0005(00)80050-6 10709854

[B61] Sacilotto N. Chouliaras K. M. Nikitenko L. L. Lu Y. W. Fritzsche M. Wallace M. D. (2016). MEF2 transcription factors are key regulators of sprouting angiogenesis. *Genes Dev.* 30 2297–2309. 10.1101/gad.290619.116 27898394 PMC5110996

[B62] Samal R. Ameling S. Wenzel K. Dhople V. Volker U. Felix S. B. (2012). OMICS-based exploration of the molecular phenotype of resident cardiac progenitor cells from adult murine heart. *J. Proteom.* 75 5304–5315. 10.1016/j.jprot.2012.06.010 22749858

[B63] Schubeler D. (2015). Function and information content of DNA methylation. *Nature* 517 321–326. 10.1038/nature14192 25592537

[B64] Sipos P. I. Crocker I. P. Hubel C. A. Baker P. N. (2010). Endothelial progenitor cells: their potential in the placental vasculature and related complications. *Placenta* 31 1–10. 10.1016/j.placenta.2009.10.006 19917514

[B65] Sipos P. I. Rens W. Schlecht H. Fan X. Wareing M. Hayward C. (2013). Uterine vasculature remodeling in human pregnancy involves functional macrochimerism by endothelial colony forming cells of fetal origin. *Stem Cells* 31 1363–1370. 10.1002/stem.1385 23554274 PMC3813980

[B66] Soliven B. Ma L. Bae H. Attali B. Sobko A. Iwase T. (2003). PDGF upregulates delayed rectifier via Src family kinases and sphingosine kinase in oligodendroglial progenitors. *Am. J. Physiol. Cell Physiol.* 284 C85–C93. 10.1152/ajpcell.00145.2002 12475761

[B67] Song X. Rui C. Meng L. Zhang R. Shen R. Ding H. (2017). Long non-coding RNA RPAIN regulates the invasion, and apoptosis of trophoblast cell lines via complement protein C1q. *Oncotarget* 8 7637–7646. 10.18632/oncotarget.13826 28032589 PMC5352349

[B68] Szklarczyk D. Morris J. H. Cook H. Kuhn M. Wyder S. Simonovic M. (2017). The STRING database in: quality-controlled protein-protein association networks, made broadly accessible. *Nucleic Acids Res.* 45 D362–D368. 10.1093/nar/gkw937 27924014 PMC5210637

[B69] Teschendorff A. E. Marabita F. Lechner M. Bartlett T. Tegner J. Gomez-Cabrero D. (2013). A beta-mixture quantile normalization method for correcting probe design bias in Illumina Infinium 450 k DNA methylation data. *Bioinformatics* 29 189–196. 10.1093/bioinformatics/bts680 23175756 PMC3546795

[B70] Tong Y. F. Wang Y. Ding Y. Y. Li J. M. Pan X. C. Lu X. L. (2017). Cyclin-dependent kinase inhibitor p21WAF1/CIP1 facilitates the development of cardiac hypertrophy. *Cell. Physiol. Biochem.* 42 1645–1656. 10.1159/000479407 28746924

[B71] Turunen M. P. Aavik E. Yla-Herttuala S. (2009). Epigenetics and atherosclerosis. *Biochim. Biophys. Acta* 1790 886–891. 10.1016/j.bbagen.2009.02.008 19233248

[B72] Urbich C. Dimmeler S. (2004). Endothelial progenitor cells functional characterization. *Trends Cardiovasc. Med.* 14 318–322. 10.1016/j.tcm.2004.10.001 15596109

[B73] Vukasinovic M. Djukic V. Stankovic P. Krejovic-Trivic S. Trivic A. Pavlovic B. (2009). Phoniatricians aspect of international statistical classification of diseases and related health problems. *Acta Chir. Iugosl.* 56 65–69. 10.2298/ACI0903065V 20218105

[B74] Wang Y. Gu Y. Zhang Y. Lewis D. F. (2004). Evidence of endothelial dysfunction in preeclampsia: decreased endothelial nitric oxide synthase expression is associated with increased cell permeability in endothelial cells from preeclampsia. *Am. J. Obstetr. Gynecol.* 190 817–824. 10.1016/j.ajog.2003.09.049 15042020

[B75] Weitzman S. A. Turk P. W. Milkowski D. H. Kozlowski K. (1994). Free radical adducts induce alterations in DNA cytosine methylation. *Proc. Natl. Acad. Sci. U.S.A.* 91 1261–1264. 10.1073/pnas.91.4.1261 8108398 PMC43137

[B76] Wu W. Bhagat T. D. Yang X. Song J. H. Cheng Y. Agarwal R. (2013). Hypomethylation of noncoding DNA regions and overexpression of the long noncoding RNA, AFAP1-AS1, in Barrett’s esophagus and esophageal adenocarcinoma. *Gastroenterology* 144 956.e4–966.e4. 10.1053/j.gastro.2013.01.019 23333711 PMC3739703

[B77] Xu Y. Ge Z. Zhang E. Zuo Q. Huang S. Yang N. (2017). The lncRNA TUG1 modulates proliferation in trophoblast cells via epigenetic suppression of RND3. *Cell Death Dis.* 8:e3104. 10.1038/cddis.2017.503 29022920 PMC5682669

[B78] Yang G. Lei Y. Inoue A. Piao L. Hu L. Jiang H. (2017). Exenatide mitigated diet-induced vascular aging and atherosclerotic plaque growth in ApoE-deficient mice under chronic stress. *Atherosclerosis* 264 1–10. 10.1016/j.atherosclerosis.2017.07.014 28734203

[B79] Yu W. Gius D. Onyango P. Muldoon-Jacobs K. Karp J. Feinberg A. P. (2008). Epigenetic silencing of tumour suppressor gene p15 by its antisense RNA. *Nature* 451 202–206. 10.1038/nature06468 18185590 PMC2743558

[B80] Yuen R. K. Penaherrera M. S. von Dadelszen P. McFadden D. E. Robinson W. P. (2010). DNA methylation profiling of human placentas reveals promoter hypomethylation of multiple genes in early-onset preeclampsia. *Eur. J. Hum. Genet.* 18 1006–1012. 10.1038/ejhg.2010.63 20442742 PMC2987406

[B81] Zeboudj L. Maitre M. Guyonnet L. Laurans L. Joffre J. Lemarie J. (2018). Selective EGF-receptor inhibition in CD4(+) T cells induces anergy and limits atherosclerosis. *J. Am. Coll. Cardiol.* 71 160–172. 10.1016/j.jacc.2017.10.084 29325640

[B82] Zhou W. Laird P. W. Shen H. (2017). Comprehensive characterization, annotation and innovative use of Infinium DNA methylation BeadChip probes. *Nucleic Acids Res.* 45:e22. 10.1093/nar/gkw967 27924034 PMC5389466

