# Supplementary material for: Preeclampsia-Associated Alteration of DNA Methylation in Fetal Endothelial Progenitor Cells
Source: Front Cell Dev Biol. 2019 Mar 19;7:32. doi: 10.3389/fcell.2019.00032 (PMC6436196; doi:10.3389/fcell.2019.00032)
Supplement: TABLE S1 — List of Gene Ontology biological processes that were significantly enriched (FDR < 0.05) in passage 5 ECFC from preeclamptic patients versus passage 5 ECFC from healthy donors. [file Data_Sheet_1.PDF]

#pathway ID	pathway description	observed gen	false discovery rate
GO.0044238	primary metabolic process	765	6.75e-16
GO.0044260	cellular macromolecule metabolic process	636	6.75e-16
GO.0043170	macromolecule metabolic process	678	1.06e-15
GO.0006996	organelle organization	322	2.97e-14
GO.0009893	positive regulation of metabolic process	374	3.68e-14
GO.0008152	metabolic process	827	4.21e-14
GO.0019222	regulation of metabolic process	593	8.94e-14
GO.0071704	organic substance metabolic process	766	8.98e-14
GO.0044237	cellular metabolic process	740	1.97e-13
GO.0060255	regulation of macromolecule metabolic process	506	1.35e-11
GO.1902589	single-organism organelle organization	230	4.78e-11
GO.0016043	cellular component organization	457	5.34e-11
GO.0071840	cellular component organization or biogenesis	466	5.34e-11
GO.0043412	macromolecule modification	301	7.78e-11
GO.0010604	positive regulation of macromolecule metabolic process	286	1.55e-10
GO.0031323	regulation of cellular metabolic process	517	2.69e-10
GO.1902680	positive regulation of RNA biosynthetic process	178	5.1e-10
GO.0006464	cellular protein modification process	284	5.29e-10
GO.0051254	positive regulation of RNA metabolic process	181	5.94e-10
GO.0080090	regulation of primary metabolic process	499	6.5e-10
GO.0048518	positive regulation of biological process	471	6.93e-10
GO.0010468	regulation of gene expression	388	9.74e-10
GO.0045893	positive regulation of transcription, DNA-templated	174	1.01e-09
GO.0051252	regulation of RNA metabolic process	347	1.44e-09
GO.0051171	regulation of nitrogen compound metabolic process	393	1.48e-09
GO.0007010	cytoskeleton organization	116	1.66e-09
GO.0009653	anatomical structure morphogenesis	233	1.81e-09
GO.0007049	cell cycle	165	2.24e-09
GO.0031325	positive regulation of cellular metabolic process	294	2.74e-09
GO.2000112	regulation of cellular macromolecule biosynthetic process	362	3.95e-09
GO.0006139	nucleobase-containing compound metabolic process	428	4.37e-09
GO.0010628	positive regulation of gene expression	194	6.42e-09
GO.0019219	regulation of nucleobase-containing compound metabolic process	366	6.78e-09
GO.1903506	regulation of nucleic acid-templated transcription	334	7.93e-09
GO.0044267	cellular protein metabolic process	337	9.14e-09
GO.0010556	regulation of macromolecule biosynthetic process	365	1,00E-08
GO.0010557	positive regulation of macromolecule biosynthetic process	187	1,00E-08
GO.0043547	positive regulation of GTPase activity	79	1,00E-08
GO.0006355	regulation of transcription, DNA-templated	333	1.09e-08
GO.2001141	regulation of RNA biosynthetic process	334	1.21e-08
GO.0019538	protein metabolic process	381	2.55e-08
GO.0009889	regulation of biosynthetic process	380	3.14e-08
GO.0006468	protein phosphorylation	110	3.52e-08
GO.0016310	phosphorylation	136	4.48e-08
GO.0007399	nervous system development	217	4.91e-08
GO.0007166	cell surface receptor signaling pathway	223	5.07e-08
GO.0031328	positive regulation of cellular biosynthetic process	194	5.07e-08
GO.0031326	regulation of cellular biosynthetic process	375	5.56e-08
GO.0045935	positive regulation of nucleobase-containing compound metabolic process	189	5.64e-08
GO.0043087	regulation of GTPase activity	82	6.35e-08
GO.0050789	regulation of biological process	773	6.4e-08
GO.0010467	gene expression	383	6.43e-08
GO.0046483	heterocycle metabolic process	434	8.17e-08
GO.0009891	positive regulation of biosynthetic process	196	8.49e-08
GO.0048522	positive regulation of cellular process	406	9.66e-08
GO.0090304	nucleic acid metabolic process	382	9.96e-08
GO.0022402	cell cycle process	131	1.05e-07
GO.0051173	positive regulation of nitrogen compound metabolic process	194	1.06e-07
GO.0044093	positive regulation of molecular function	189	1.24e-07
GO.0065007	biological regulation	793	1.47e-07
GO.0006725	cellular aromatic compound metabolic process	431	2.21e-07
GO.0043933	macromolecular complex subunit organization	224	2.21e-07
GO.0048468	cell development	181	2.21e-07
GO.0009987	cellular process	969	2.31e-07
GO.0016070	RNA metabolic process	342	2.31e-07
GO.0030030	cell projection organization	124	3.2e-07
GO.0006807	nitrogen compound metabolic process	494	3.46e-07
GO.0006796	phosphate-containing compound metabolic process	196	3.48e-07
GO.0051345	positive regulation of hydrolase activity	109	3.48e-07
GO.1901360	organic cyclic compound metabolic process	447	3.67e-07
GO.0043085	positive regulation of catalytic activity	163	4.41e-07
GO.0048519	negative regulation of biological process	393	4.41e-07
GO.0065009	regulation of molecular function	270	4.42e-07
GO.0000278	mitotic cell cycle	108	6.49e-07
GO.0006793	phosphorus metabolic process	198	8.05e-07
GO.0048666	neuron development	105	8.05e-07
GO.0006357	regulation of transcription from RNA polymerase II promoter	183	9.63e-07
GO.0034641	cellular nitrogen compound metabolic process	465	1.04e-06
GO.0050794	regulation of cellular process	739	1.1e-06
GO.0022008	neurogenesis	159	1.28e-06
GO.0034645	cellular macromolecule biosynthetic process	351	1.66e-06
GO.0048523	negative regulation of cellular process	365	1.66e-06
GO.0006351	transcription, DNA-templated	268	2.29e-06

GO.0051336	regulation of hydrolase activity	145	2.42e-06
GO.0000902	cell morphogenesis	116	2.76e-06
GO.0032502	developmental process	421	2.78e-06
GO.0048699	generation of neurons	151	2.81e-06
GO.0071310	cellular response to organic substance	196	2.91e-06
GO.0000226	microtubule cytoskeleton organization	55	3.12e-06
GO.0000904	cell morphogenesis involved in differentiation	89	3.2e-06
GO.0051726	regulation of cell cycle	116	4.3e-06
GO.0044772	mitotic cell cycle phase transition	53	5.09e-06
GO.0031399	regulation of protein modification process	172	5.4e-06
GO.1903047	mitotic cell cycle process	96	5.69e-06
GO.0009059	macromolecule biosynthetic process	353	6.45e-06
GO.0032990	cell part morphogenesis	98	6.82e-06
GO.0048858	cell projection morphogenesis	96	6.82e-06
GO.0031175	neuron projection development	88	6.9e-06
GO.0032989	cellular component morphogenesis	122	6.9e-06
GO.0022607	cellular component assembly	189	7.48e-06
GO.0044085	cellular component biogenesis	203	8.21e-06
GO.0050790	regulation of catalytic activity	223	8.58e-06
GO.0016192	vesicle-mediated transport	132	8.8e-06
GO.0048812	neuron projection morphogenesis	76	8.82e-06
GO.0051128	regulation of cellular component organization	218	9.39e-06
GO.0007017	microtubule-based process	70	9.65e-06
GO.0045944	positive regulation of transcription from RNA polymerase II promoter	119	1.19e-05
GO.0044767	single-organism developmental process	413	1.25e-05
GO.0040012	regulation of locomotion	90	1.41e-05
GO.0051276	chromosome organization	107	1.41e-05
GO.0048856	anatomical structure development	373	1.45e-05
GO.0030182	neuron differentiation	116	1.51e-05
GO.0032774	RNA biosynthetic process	273	1.77e-05
GO.0061564	axon development	71	2.27e-05
GO.0007409	axonogenesis	69	2.29e-05
GO.0048667	cell morphogenesis involved in neuron differentiation	73	2.76e-05
GO.0051246	regulation of protein metabolic process	238	2.76e-05
GO.0032268	regulation of cellular protein metabolic process	225	2.94e-05
GO.0070887	cellular response to chemical stimulus	226	3.53e-05
GO.0010605	negative regulation of macromolecule metabolic process	220	4.63e-05
GO.0009892	negative regulation of metabolic process	242	5.07e-05
GO.0007169	transmembrane receptor protein tyrosine kinase signaling pathway	88	5.39e-05
GO.0007167	enzyme linked receptor protein signaling pathway	109	5.44e-05
GO.0032879	regulation of localization	224	5.84e-05
GO.0035556	intracellular signal transduction	187	5.84e-05
GO.0034654	nucleobase-containing compound biosynthetic process	293	6.59e-05
GO.0007275	multicellular organismal development	366	7.25e-05
GO.0006325	chromatin organization	82	7.45e-05
GO.0009790	embryo development	111	8.11e-05
GO.0019438	aromatic compound biosynthetic process	298	8.11e-05
GO.0034333	adherens junction assembly	13	9.27e-05
GO.0051017	actin filament bundle assembly	14	0.000123
GO.0016568	chromatin modification	71	0.000127
GO.0007411	axon guidance	57	0.000131
GO.0009966	regulation of signal transduction	231	0.000135
GO.0044249	cellular biosynthetic process	406	0.000146
GO.2000145	regulation of cell motility	81	0.000151
GO.0048583	regulation of response to stimulus	304	0.00016
GO.0072358	cardiovascular system development	95	0.000161
GO.0072359	circulatory system development	95	0.000161
GO.0051270	regulation of cellular component movement	88	0.000169
GO.0035295	tube development	75	0.000172
GO.0009968	negative regulation of signal transduction	118	0.000173
GO.0048869	cellular developmental process	298	0.000174
GO.0051247	positive regulation of protein metabolic process	147	0.000195
GO.0048731	system development	321	0.000215
GO.0018130	heterocycle biosynthetic process	294	0.000224
GO.0050793	regulation of developmental process	204	0.000257
GO.0007420	brain development	81	0.000266
GO.0044763	single-organism cellular process	787	0.00027
GO.0051129	negative regulation of cellular component organization	72	0.000272
GO.0033554	cellular response to stress	166	0.000294
GO.0010033	response to organic substance	234	0.000301
GO.0051130	positive regulation of cellular component organization	125	0.000331
GO.0051641	cellular localization	204	0.000416
GO.0000122	negative regulation of transcription from RNA polymerase II promoter	90	0.000452
GO.0016569	covalent chromatin modification	48	0.000475
GO.0030900	forebrain development	50	0.000478
GO.1901576	organic substance biosynthetic process	407	0.000478
GO.0051338	regulation of transferase activity	101	0.000506
GO.1901362	organic cyclic compound biosynthetic process	301	0.000527
GO.0009058	biosynthetic process	413	0.000562
GO.0060322	head development	84	0.000562
GO.0023051	regulation of signaling	251	0.000573
GO.0070848	response to growth factor	80	0.000604
GO.0071363	cellular response to growth factor stimulus	78	0.000623
GO.0044271	cellular nitrogen compound biosynthetic process	316	0.000647

GO.0010629	negative regulation of gene expression	147	0.000652
GO.0031401	positive regulation of protein modification process	114	0.000708
GO.0001841	neural tube formation	23	0.000712
GO.1903827	regulation of cellular protein localization	65	0.000712
GO.0016570	histone modification	47	0.000732
GO.0018193	peptidyl-amino acid modification	98	0.000732
GO.0009887	organ morphogenesis	99	0.000757
GO.0030036	actin cytoskeleton organization	53	0.000763
GO.0035239	tube morphogenesis	51	0.000777
GO.0038093	Fc receptor signaling pathway	37	0.000805
GO.0051347	positive regulation of transferase activity	71	0.000805
GO.0031400	negative regulation of protein modification process	72	0.000816
GO.0010646	regulation of cell communication	261	0.000841
GO.0023057	negative regulation of signaling	124	0.000864
GO.0032270	positive regulation of cellular protein metabolic process	135	0.000934
GO.0010256	endomembrane system organization	57	0.000989
GO.0030154	cell differentiation	280	0.00103
GO.0031324	negative regulation of cellular metabolic process	212	0.00106
GO.0010648	negative regulation of cell communication	124	0.00125
GO.0043632	modification-dependent macromolecule catabolic process	59	0.00131
GO.0080134	regulation of response to stress	144	0.00136
GO.0003007	heart morphogenesis	35	0.00143
GO.0001843	neural tube closure	20	0.00144
GO.0051239	regulation of multicellular organismal process	220	0.00144
GO.0033043	regulation of organelle organization	117	0.00147
GO.0043549	regulation of kinase activity	85	0.00148
GO.0051716	cellular response to stimulus	489	0.00148
GO.0048585	negative regulation of response to stimulus	135	0.00149
GO.0019941	modification-dependent protein catabolic process	58	0.00159
GO.0030334	regulation of cell migration	73	0.00159
GO.0043009	chordate embryonic development	75	0.0016
GO.0051179	localization	369	0.00164
GO.0008543	fibroblast growth factor receptor signaling pathway	29	0.00169
GO.0008150	biological_process	955	0.0017
GO.0006511	ubiquitin-dependent protein catabolic process	57	0.00184
GO.0033674	positive regulation of kinase activity	59	0.00208
GO.0048285	organelle fission	59	0.00208
GO.0007154	cell communication	424	0.00215
GO.0060562	epithelial tube morphogenesis	45	0.00221
GO.0021915	neural tube development	28	0.00238
GO.0000086	G2/M transition of mitotic cell cycle	26	0.00242
GO.0040029	regulation of gene expression, epigenetic	35	0.0026
GO.0017015	regulation of transforming growth factor beta receptor signaling pathway	21	0.00272
GO.0014020	primary neural tube formation	20	0.00293
GO.0048598	embryonic morphogenesis	68	0.00318
GO.0019220	regulation of phosphate metabolic process	149	0.00335
GO.0007507	heart development	57	0.0034
GO.0060627	regulation of vesicle-mediated transport	51	0.00341
GO.0030029	actin filament-based process	54	0.00353
GO.0051174	regulation of phosphorus metabolic process	150	0.00355
GO.0000082	G1/S transition of mitotic cell cycle	29	0.00378
GO.0001838	embryonic epithelial tube formation	24	0.00378
GO.0010639	negative regulation of organelle organization	41	0.00378
GO.0048011	neurotrophin TRK receptor signaling pathway	41	0.00378
GO.0030111	regulation of Wnt signaling pathway	42	0.004
GO.0051172	negative regulation of nitrogen compound metabolic process	141	0.00423
GO.0000280	nuclear division	55	0.00425
GO.0044699	single-organism process	807	0.00425
GO.0030163	protein catabolic process	66	0.00441
GO.0007229	integrin-mediated signaling pathway	17	0.00443
GO.0045860	positive regulation of protein kinase activity	55	0.00448
GO.0006950	response to stress	299	0.00453
GO.0051649	establishment of localization in cell	168	0.00458
GO.0045859	regulation of protein kinase activity	78	0.00472
GO.0042325	regulation of phosphorylation	128	0.00479
GO.0023052	signaling	412	0.0048
GO.0070201	regulation of establishment of protein localization	88	0.00482
GO.0007417	central nervous system development	93	0.00486
GO.0033044	regulation of chromosome organization	32	0.00501
GO.0065003	macromolecular complex assembly	120	0.00505
GO.0042176	regulation of protein catabolic process	54	0.0051
GO.0034332	adherens junction organization	16	0.00515
GO.0044700	single organism signaling	411	0.00558
GO.0044344	cellular response to fibroblast growth factor stimulus	30	0.00559
GO.0032880	regulation of protein localization	98	0.0056
GO.0060341	regulation of cellular localization	121	0.0056
GO.0006367	transcription initiation from RNA polymerase II promoter	37	0.0057
GO.0038095	Fc-epsilon receptor signaling pathway	28	0.00615
GO.0051348	negative regulation of transferase activity	44	0.00649
GO.0051253	negative regulation of RNA metabolic process	121	0.00668
GO.0007346	regulation of mitotic cell cycle	57	0.0067
GO.0035148	tube formation	25	0.00687
GO.0033036	macromolecule localization	189	0.0069
GO.0071495	cellular response to endogenous stimulus	106	0.0069

GO.0030512	negative regulation of transforming growth factor beta receptor signaling pathway	16	0.00696
GO.0045934	negative regulation of nucleobase-containing compound metabolic process	131	0.007
GO.0002009	morphogenesis of an epithelium	55	0.00714
GO.0032386	regulation of intracellular transport	67	0.00726
GO.0051603	proteolysis involved in cellular protein catabolic process	59	0.00746
GO.0045892	negative regulation of transcription, DNA-templated	115	0.00755
GO.0046777	protein autophosphorylation	30	0.00775
GO.0002764	immune response-regulating signaling pathway	58	0.00776
GO.0050896	response to stimulus	549	0.0078
GO.0051301	cell division	58	0.00817
GO.0001932	regulation of protein phosphorylation	119	0.00832
GO.0006352	DNA-templated transcription, initiation	40	0.00833
GO.0045787	positive regulation of cell cycle	42	0.00856
GO.0007165	signal transduction	386	0.00867
GO.0033135	regulation of peptidyl-serine phosphorylation	21	0.00873
GO.0022011	myelination in peripheral nervous system	8	0.00882
GO.0033157	regulation of intracellular protein transport	47	0.00886
GO.2000113	negative regulation of cellular macromolecule biosynthetic process	127	0.00886
GO.0007264	small GTPase mediated signal transduction	75	0.00932
GO.0006396	RNA processing	79	0.0097
GO.0006461	protein complex assembly	100	0.0105
GO.0044257	cellular protein catabolic process	60	0.0105
GO.0070271	protein complex biogenesis	100	0.0105
GO.0071902	positive regulation of protein serine/threonine kinase activity	38	0.0105
GO.0006928	movement of cell or subcellular component	130	0.0108
GO.0031647	regulation of protein stability	28	0.0111
GO.1903507	negative regulation of nucleic acid-templated transcription	115	0.0111
GO.0001952	regulation of cell-matrix adhesion	17	0.0113
GO.0016265	death	114	0.0113
GO.0050767	regulation of neurogenesis	67	0.0113
GO.0010564	regulation of cell cycle process	62	0.0114
GO.2000026	regulation of multicellular organismal development	147	0.0114
GO.0043161	proteasome-mediated ubiquitin-dependent protein catabolic process	41	0.0118
GO.0046330	positive regulation of JNK cascade	20	0.012
GO.0060429	epithelium development	101	0.0125
GO.0006915	apoptotic process	109	0.0126
GO.0048646	anatomical structure formation involved in morphogenesis	102	0.0126
GO.0071822	protein complex subunit organization	138	0.0129
GO.0006897	endocytosis	59	0.0131
GO.0032006	regulation of TOR signaling	14	0.0131
GO.0008219	cell death	113	0.0134
GO.0038127	ERBB signaling pathway	31	0.0134
GO.0046907	intracellular transport	128	0.0134
GO.0043393	regulation of protein binding	25	0.014
GO.1903050	regulation of proteolysis involved in cellular protein catabolic process	39	0.014
GO.0060828	regulation of canonical Wnt signaling pathway	33	0.0143
GO.0016331	morphogenesis of embryonic epithelium	25	0.0153
GO.1902275	regulation of chromatin organization	23	0.0153
GO.0001933	negative regulation of protein phosphorylation	44	0.0164
GO.0030178	negative regulation of Wnt signaling pathway	28	0.0166
GO.0012501	programmed cell death	109	0.0169
GO.0042327	positive regulation of phosphorylation	88	0.017
GO.0001934	positive regulation of protein phosphorylation	85	0.0174
GO.0051223	regulation of protein transport	79	0.0181
GO.0008104	protein localization	158	0.0183
GO.0044764	multi-organism cellular process	78	0.0183
GO.1903308	regulation of chromatin modification	22	0.0193
GO.0044710	single-organism metabolic process	349	0.0194
GO.0051248	negative regulation of protein metabolic process	104	0.0197
GO.0009628	response to abiotic stimulus	111	0.0206
GO.0010762	regulation of fibroblast migration	8	0.0206
GO.0090004	positive regulation of establishment of protein localization to plasma membrane	9	0.0207
GO.0007173	epidermal growth factor receptor signaling pathway	30	0.0209
GO.0051271	negative regulation of cellular component movement	31	0.0209
GO.0003151	outflow tract morphogenesis	13	0.0211
GO.0002218	activation of innate immune response	34	0.0213
GO.0002768	immune response-regulating cell surface receptor signaling pathway	47	0.0213
GO.1903829	positive regulation of cellular protein localization	41	0.0213
GO.0007050	cell cycle arrest	23	0.0218
GO.0032269	negative regulation of cellular protein metabolic process	98	0.0218
GO.0033673	negative regulation of kinase activity	31	0.0221
GO.0048729	tissue morphogenesis	65	0.0225
GO.0034501	protein localization to kinetochore	5	0.0227
GO.2000392	regulation of lamellipodium morphogenesis	5	0.0227
GO.0016032	viral process	77	0.023
GO.0044265	cellular macromolecule catabolic process	82	0.0244
GO.0045936	negative regulation of phosphate metabolic process	58	0.0249
GO.0014037	Schwann cell differentiation	9	0.0255
GO.0021987	cerebral cortex development	18	0.0256
GO.0014044	Schwann cell development	8	0.0259
GO.0031098	stress-activated protein kinase signaling cascade	23	0.0259
GO.0032874	positive regulation of stress-activated MAPK cascade	21	0.0259
GO.0045595	regulation of cell differentiation	139	0.0259
GO.0051895	negative regulation of focal adhesion assembly	6	0.0259

GO.0002758	innate immune response-activating signal transduction	33	0.026
GO.0010558	negative regulation of macromolecule biosynthetic process	128	0.0264
GO.0006469	negative regulation of protein kinase activity	29	0.0275
GO.0002244	hematopoietic progenitor cell differentiation	19	0.0276
GO.0060284	regulation of cell development	80	0.0277
GO.0001501	skeletal system development	53	0.0286
GO.0046328	regulation of JNK cascade	24	0.0286
GO.0051091	positive regulation of sequence-specific DNA binding transcription factor activity	31	0.0286
GO.1901990	regulation of mitotic cell cycle phase transition	34	0.0288
GO.0009057	macromolecule catabolic process	93	0.029
GO.0090101	negative regulation of transmembrane receptor protein serine/threonine kinase signaling pathway	19	0.0303
GO.0042787	protein ubiquitination involved in ubiquitin-dependent protein catabolic process	15	0.0308
GO.0043506	regulation of JUN kinase activity	15	0.0308
GO.0042326	negative regulation of phosphorylation	46	0.0327
GO.1901699	cellular response to nitrogen compound	60	0.0327
GO.0035194	posttranscriptional gene silencing by RNA	10	0.0334
GO.0009890	negative regulation of biosynthetic process	134	0.0348
GO.0006366	transcription from RNA polymerase II promoter	82	0.0352
GO.0043149	stress fiber assembly	5	0.0355
GO.1902531	regulation of intracellular signal transduction	131	0.036
GO.0043507	positive regulation of JUN kinase activity	13	0.0364
GO.0034330	cell junction organization	27	0.0367
GO.0040011	locomotion	115	0.0367
GO.1903729	regulation of plasma membrane organization	14	0.0381
GO.0016055	Wnt signaling pathway	32	0.0388
GO.0071230	cellular response to amino acid stimulus	11	0.0397
GO.0090003	regulation of establishment of protein localization to plasma membrane	11	0.0397
GO.0001702	gastrulation with mouth forming second	8	0.0405
GO.0090073	positive regulation of protein homodimerization activity	4	0.0405
GO.2000394	positive regulation of lamellipodium morphogenesis	4	0.0405
GO.1904031	positive regulation of cyclin-dependent protein kinase activity	7	0.0408
GO.1903076	regulation of protein localization to plasma membrane	13	0.041
GO.0034329	cell junction assembly	24	0.0421
GO.0051049	regulation of transport	158	0.0421
GO.0023014	signal transduction by protein phosphorylation	33	0.0452
GO.0090630	activation of GTPase activity	9	0.0452
GO.1901987	regulation of cell cycle phase transition	35	0.0452
GO.0061024	membrane organization	88	0.0463
GO.1903320	regulation of protein modification by small protein conjugation or removal	34	0.0466
GO.0031327	negative regulation of cellular biosynthetic process	131	0.0468
GO.0010608	posttranscriptional regulation of gene expression	42	0.047
GO.0033138	positive regulation of peptidyl-serine phosphorylation	16	0.0483
GO.0007020	microtubule nucleation	6	0.0486
GO.0043496	regulation of protein homodimerization activity	6	0.0486
GO.0006508	proteolysis	106	0.0489
GO.0071900	regulation of protein serine/threonine kinase activity	50	0.0489
GO.0001953	negative regulation of cell-matrix adhesion	8	0.0496
