# Supplementary material for: Preeclampsia-Associated Alteration of DNA Methylation in Fetal Endothelial Progenitor Cells
Source: Front Cell Dev Biol. 2019 Mar 19;7:32. doi: 10.3389/fcell.2019.00032 (PMC6436196; doi:10.3389/fcell.2019.00032)
Supplement: TABLE S2 — List of Gene Ontology biological processes that were significantly enriched (FDR < 0.05) in passage 3 ECFC from preeclamptic patients versus passage 3 ECFC from healthy donors. [file Data_Sheet_2.PDF]

| #pathway ID | pathway description | observed gene count | false discovery rate |
|---|---|---|---|
| GO.0044238 | primary metabolic process | 431 | 8.4e-09 |
| GO.0044260 | cellular macromolecule metabolic process | 356 | 2.47e-08 |
| GO.0043170 | macromolecule metabolic process | 376 | 1.28e-07 |
| GO.0007166 | cell surface receptor signaling pathway | 141 | 3.21e-07 |
| GO.0006468 | protein phosphorylation | 72 | 5.77e-07 |
| GO.0007049 | cell cycle | 102 | 5.77e-07 |
| GO.0000278 | mitotic cell cycle | 73 | 7.76e-07 |
| GO.0022402 | cell cycle process | 85 | 7.76e-07 |
| GO.0071704 | organic substance metabolic process | 425 | 7.76e-07 |
| GO.0008152 | metabolic process | 456 | 1.15e-06 |
| GO.0016310 | phosphorylation | 86 | 1.4e-06 |
| GO.0007010 | cytoskeleton organization | 71 | 1.51e-06 |
| GO.0019222 | regulation of metabolic process | 326 | 1.95e-06 |
| GO.0044237 | cellular metabolic process | 407 | 3.36e-06 |
| GO.0006996 | organelle organization | 173 | 4.15e-06 |
| GO.0009893 | positive regulation of metabolic process | 202 | 4.15e-06 |
| GO.0044772 | mitotic cell cycle phase transition | 38 | 4.15e-06 |
| GO.0060255 | regulation of macromolecule metabolic process | 282 | 4.15e-06 |
| GO.1903047 | mitotic cell cycle process | 65 | 4.15e-06 |
| GO.0006796 | phosphate-containing compound metabolic process | 121 | 4.63e-06 |
| GO.0016043 | cellular component organization | 256 | 4.63e-06 |
| GO.0048518 | positive regulation of biological process | 268 | 4.63e-06 |
| GO.0071840 | cellular component organization or biogenesis | 261 | 4.63e-06 |
| GO.0006793 | phosphorus metabolic process | 122 | 8.93e-06 |
| GO.0006464 | cellular protein modification process | 161 | 9.42e-06 |
| GO.0050789 | regulation of biological process | 441 | 1.31e-05 |
| GO.0065007 | biological regulation | 453 | 1.69e-05 |
| GO.0007165 | signal transduction | 248 | 1.89e-05 |
| GO.0007154 | cell communication | 266 | 1.92e-05 |
| GO.0044700 | single organism signaling | 261 | 1.96e-05 |
| GO.0043412 | macromolecule modification | 166 | 2.24e-05 |
| GO.0035556 | intracellular signal transduction | 119 | 2.97e-05 |
| GO.0048522 | positive regulation of cellular process | 234 | 3.32e-05 |
| GO.0031323 | regulation of cellular metabolic process | 285 | 4.76e-05 |
| GO.0071310 | cellular response to organic substance | 119 | 4.87e-05 |
| GO.0000226 | microtubule cytoskeleton organization | 36 | 5.45e-05 |
| GO.0050794 | regulation of cellular process | 423 | 5.54e-05 |
| GO.0044267 | cellular protein metabolic process | 190 | 6.15e-05 |
| GO.0007167 | enzyme linked receptor protein signaling pathway | 71 | 6.33e-05 |
| GO.0007169 | transmembrane receptor protein tyrosine kinase signaling pathway | 58 | 7.66e-05 |
| GO.0009987 | cellular process | 546 | 7.66e-05 |
| GO.0019538 | protein metabolic process | 215 | 8.87e-05 |
| GO.0051254 | positive regulation of RNA metabolic process | 99 | 9.69e-05 |
| GO.1902680 | positive regulation of RNA biosynthetic process | 97 | 0.0001 |
| GO.0043085 | positive regulation of catalytic activity | 96 | 0.000108 |
| GO.0044093 | positive regulation of molecular function | 109 | 0.000122 |
| GO.0010604 | positive regulation of macromolecule metabolic process | 154 | 0.000141 |
| GO.0045893 | positive regulation of transcription, DNA-templated | 95 | 0.000141 |
| GO.2000112 | regulation of cellular macromolecule biosynthetic process | 200 | 0.000156 |
| GO.1903506 | regulation of nucleic acid-templated transcription | 185 | 0.000192 |
| GO.0051171 | regulation of nitrogen compound metabolic process | 215 | 0.000199 |
| GO.0010468 | regulation of gene expression | 211 | 0.00024 |
| GO.1902589 | single-organism organelle organization | 121 | 0.00024 |
| GO.0051726 | regulation of cell cycle | 70 | 0.000241 |
| GO.0080090 | regulation of primary metabolic process | 271 | 0.000265 |
| GO.0006355 | regulation of transcription, DNA-templated | 184 | 0.000268 |
| GO.0010556 | regulation of macromolecule biosynthetic process | 201 | 0.000328 |
| GO.0009653 | anatomical structure morphogenesis | 126 | 0.000341 |
| GO.0051252 | regulation of RNA metabolic process | 188 | 0.000341 |
| GO.0051716 | cellular response to stimulus | 293 | 0.000341 |
| GO.2001141 | regulation of RNA biosynthetic process | 184 | 0.000341 |
| GO.0019219 | regulation of nucleobase-containing compound metabolic process | 200 | 0.00044 |
| GO.0048523 | negative regulation of cellular process | 208 | 0.000464 |
| GO.0007017 | microtubule-based process | 43 | 0.000479 |
| GO.0031325 | positive regulation of cellular metabolic process | 159 | 0.000479 |
| GO.0010628 | positive regulation of gene expression | 105 | 0.000538 |
| GO.0051345 | positive regulation of hydrolase activity | 62 | 0.000601 |
| GO.0030900 | forebrain development | 34 | 0.000608 |
| GO.0000086 | G2/M transition of mitotic cell cycle | 20 | 0.000612 |
| GO.0006139 | nucleobase-containing compound metabolic process | 232 | 0.00067 |
| GO.0030030 | cell projection organization | 70 | 0.00067 |
| GO.0051128 | regulation of cellular component organization | 127 | 0.00067 |
| GO.0007420 | brain development | 52 | 0.000694 |
| GO.0010557 | positive regulation of macromolecule biosynthetic process | 101 | 0.000712 |
| GO.0070887 | cellular response to chemical stimulus | 133 | 0.000814 |

| | | | |
|---|---|---|---|
| GO.0065009 | regulation of molecular function | 151 | 0.000906 |
| GO.0034645 | cellular macromolecule biosynthetic process | 198 | 0.00091 |
| GO.0031175 | neuron projection development | 52 | 0.000945 |
| GO.0050896 | response to stimulus | 328 | 0.000989 |
| GO.0060322 | head development | 54 | 0.00102 |
| GO.0045935 | positive regulation of nucleobase-containing compound metabolic process | 103 | 0.0011 |
| GO.0048583 | regulation of response to stimulus | 179 | 0.0011 |
| GO.0051173 | positive regulation of nitrogen compound metabolic process | 106 | 0.00142 |
| GO.0031328 | positive regulation of cellular biosynthetic process | 105 | 0.00143 |
| GO.0007399 | nervous system development | 117 | 0.00177 |
| GO.0031399 | regulation of protein modification process | 98 | 0.00177 |
| GO.0043547 | positive regulation of GTPase activity | 41 | 0.00177 |
| GO.0010467 | gene expression | 208 | 0.00206 |
| GO.0060828 | regulation of canonical Wnt signaling pathway | 25 | 0.0022 |
| GO.0031326 | regulation of cellular biosynthetic process | 203 | 0.00226 |
| GO.0016070 | RNA metabolic process | 187 | 0.0024 |
| GO.0048519 | negative regulation of biological process | 216 | 0.00248 |
| GO.0048666 | neuron development | 58 | 0.00248 |
| GO.0030182 | neuron differentiation | 67 | 0.00252 |
| GO.0051336 | regulation of hydrolase activity | 81 | 0.00274 |
| GO.0009889 | regulation of biosynthetic process | 204 | 0.00282 |
| GO.0050790 | regulation of catalytic activity | 126 | 0.00282 |
| GO.0090304 | nucleic acid metabolic process | 207 | 0.00282 |
| GO.0009891 | positive regulation of biosynthetic process | 105 | 0.00291 |
| GO.0010646 | regulation of cell communication | 155 | 0.00291 |
| GO.0044763 | single-organism cellular process | 447 | 0.00294 |
| GO.0048699 | generation of neurons | 84 | 0.00338 |
| GO.0009966 | regulation of signal transduction | 134 | 0.00358 |
| GO.0009059 | macromolecule biosynthetic process | 197 | 0.00361 |
| GO.0007417 | central nervous system development | 60 | 0.00366 |
| GO.0002758 | innate immune response-activating signal transduction | 25 | 0.00368 |
| GO.0006351 | transcription, DNA-templated | 148 | 0.00385 |
| GO.0006357 | regulation of transcription from RNA polymerase II promoter | 100 | 0.00385 |
| GO.0046483 | heterocycle metabolic process | 234 | 0.00385 |
| GO.0043393 | regulation of protein binding | 19 | 0.00391 |
| GO.0022008 | neurogenesis | 87 | 0.00409 |
| GO.0071495 | cellular response to endogenous stimulus | 68 | 0.00409 |
| GO.0048812 | neuron projection morphogenesis | 43 | 0.00417 |
| GO.0032879 | regulation of localization | 128 | 0.0044 |
| GO.0050793 | regulation of developmental process | 119 | 0.00455 |
| GO.0048285 | organelle fission | 38 | 0.00487 |
| GO.0010033 | response to organic substance | 136 | 0.00501 |
| GO.0000904 | cell morphogenesis involved in differentiation | 49 | 0.00502 |
| GO.0072358 | cardiovascular system development | 56 | 0.00538 |
| GO.0072359 | circulatory system development | 56 | 0.00538 |
| GO.0006807 | nitrogen compound metabolic process | 268 | 0.0054 |
| GO.0032386 | regulation of intracellular transport | 44 | 0.0059 |
| GO.0000280 | nuclear division | 36 | 0.00609 |
| GO.0043496 | regulation of protein homodimerization activity | 6 | 0.00609 |
| GO.0030111 | regulation of Wnt signaling pathway | 28 | 0.00623 |
| GO.0023051 | regulation of signaling | 146 | 0.00629 |
| GO.0006725 | cellular aromatic compound metabolic process | 232 | 0.0063 |
| GO.0048858 | cell projection morphogenesis | 53 | 0.00653 |
| GO.0002223 | stimulatory C-type lectin receptor signaling pathway | 16 | 0.00659 |
| GO.0051239 | regulation of multicellular organismal process | 130 | 0.00659 |
| GO.0032774 | RNA biosynthetic process | 152 | 0.00697 |
| GO.0060627 | regulation of vesicle-mediated transport | 33 | 0.00758 |
| GO.1901360 | organic cyclic compound metabolic process | 241 | 0.0076 |
| GO.0071363 | cellular response to growth factor stimulus | 47 | 0.00776 |
| GO.0080134 | regulation of response to stress | 86 | 0.00779 |
| GO.0070848 | response to growth factor | 48 | 0.00829 |
| GO.0008543 | fibroblast growth factor receptor signaling pathway | 19 | 0.00835 |
| GO.0048468 | cell development | 95 | 0.00865 |
| GO.0048667 | cell morphogenesis involved in neuron differentiation | 41 | 0.00865 |
| GO.0034641 | cellular nitrogen compound metabolic process | 252 | 0.00882 |
| GO.0007346 | regulation of mitotic cell cycle | 37 | 0.00957 |
| GO.0043087 | regulation of GTPase activity | 41 | 0.00963 |
| GO.0038093 | Fc receptor signaling pathway | 23 | 0.0101 |
| GO.0090073 | positive regulation of protein homodimerization activity | 4 | 0.0106 |
| GO.0044708 | single-organism behavior | 33 | 0.011 |
| GO.0038095 | Fc-epsilon receptor signaling pathway | 19 | 0.0117 |
| GO.0045944 | positive regulation of transcription from RNA polymerase II promoter | 65 | 0.0118 |
| GO.0003007 | heart morphogenesis | 22 | 0.0123 |
| GO.0007409 | axonogenesis | 38 | 0.0128 |
| GO.0023014 | signal transduction by protein phosphorylation | 24 | 0.0128 |
| GO.0035295 | tube development | 43 | 0.0133 |

| | | | |
|---|---|---|---|
| GO.0044344 | cellular response to fibroblast growth factor stimulus | 20 | 0.0133 |
| GO.0061564 | axon development | 39 | 0.0133 |
| GO.0000082 | G1/S transition of mitotic cell cycle | 19 | 0.014 |
| GO.0050767 | regulation of neurogenesis | 43 | 0.0141 |
| GO.0060284 | regulation of cell development | 52 | 0.0153 |
| GO.0002764 | immune response-regulating signaling pathway | 37 | 0.0154 |
| GO.0031349 | positive regulation of defense response | 31 | 0.0163 |
| GO.0030177 | positive regulation of Wnt signaling pathway | 18 | 0.0168 |
| GO.0000902 | cell morphogenesis | 61 | 0.0171 |
| GO.0051174 | regulation of phosphorus metabolic process | 89 | 0.0171 |
| GO.0031401 | positive regulation of protein modification process | 66 | 0.0175 |
| GO.0003139 | secondary heart field specification | 4 | 0.0177 |
| GO.0019220 | regulation of phosphate metabolic process | 88 | 0.0192 |
| GO.0032268 | regulation of cellular protein metabolic process | 123 | 0.0192 |
| GO.0007507 | heart development | 35 | 0.0198 |
| GO.1903827 | regulation of cellular protein localization | 38 | 0.0198 |
| GO.0051129 | negative regulation of cellular component organization | 41 | 0.02 |
| GO.2000026 | regulation of multicellular organismal development | 89 | 0.0205 |
| GO.0010647 | positive regulation of cell communication | 91 | 0.0207 |
| GO.0002768 | immune response-regulating cell surface receptor signaling pathway | 31 | 0.0221 |
| GO.0033157 | regulation of intracellular protein transport | 30 | 0.0221 |
| GO.0048584 | positive regulation of response to stimulus | 106 | 0.0221 |
| GO.0051960 | regulation of nervous system development | 47 | 0.0221 |
| GO.0034654 | nucleobase-containing compound biosynthetic process | 161 | 0.0234 |
| GO.0042325 | regulation of phosphorylation | 76 | 0.0244 |
| GO.0033554 | cellular response to stress | 93 | 0.0248 |
| GO.0021987 | cerebral cortex development | 13 | 0.0257 |
| GO.0030163 | protein catabolic process | 40 | 0.0269 |
| GO.0007610 | behavior | 38 | 0.0284 |
| GO.0032989 | cellular component morphogenesis | 64 | 0.0284 |
| GO.0051130 | positive regulation of cellular component organization | 70 | 0.0284 |
| GO.0051301 | cell division | 36 | 0.0284 |
| GO.1901699 | cellular response to nitrogen compound | 39 | 0.0284 |
| GO.0008150 | biological_process | 534 | 0.0299 |
| GO.0031333 | negative regulation of protein complex assembly | 13 | 0.0299 |
| GO.0044767 | single-organism developmental process | 223 | 0.0299 |
| GO.0051049 | regulation of transport | 97 | 0.0306 |
| GO.0010648 | negative regulation of cell communication | 71 | 0.0308 |
| GO.0009967 | positive regulation of signal transduction | 77 | 0.0314 |
| GO.0090263 | positive regulation of canonical Wnt signaling pathway | 15 | 0.0314 |
| GO.0001505 | regulation of neurotransmitter levels | 16 | 0.0322 |
| GO.0001932 | regulation of protein phosphorylation | 71 | 0.0326 |
| GO.0006367 | transcription initiation from RNA polymerase II promoter | 23 | 0.0333 |
| GO.0032502 | developmental process | 224 | 0.0338 |
| GO.0007411 | axon guidance | 31 | 0.0356 |
| GO.0044249 | cellular biosynthetic process | 223 | 0.0362 |
| GO.0002757 | immune response-activating signal transduction | 30 | 0.0375 |
| GO.0023057 | negative regulation of signaling | 70 | 0.0375 |
| GO.0044085 | cellular component biogenesis | 107 | 0.0375 |
| GO.0034333 | adherens junction assembly | 7 | 0.0377 |
| GO.0023056 | positive regulation of signaling | 83 | 0.0387 |
| GO.0060341 | regulation of cellular localization | 71 | 0.0389 |
| GO.0002726 | positive regulation of T cell cytokine production | 4 | 0.0398 |
| GO.0019438 | aromatic compound biosynthetic process | 162 | 0.0402 |
| GO.0051641 | cellular localization | 113 | 0.0402 |
| GO.0032270 | positive regulation of cellular protein metabolic process | 76 | 0.0404 |
| GO.0045595 | regulation of cell differentiation | 84 | 0.041 |
| GO.0051347 | positive regulation of transferase activity | 40 | 0.0435 |
| GO.0090630 | activation of GTPase activity | 7 | 0.0435 |
| GO.0043933 | macromolecular complex subunit organization | 113 | 0.0448 |
| GO.0009968 | negative regulation of signal transduction | 64 | 0.0475 |
| GO.0051098 | regulation of binding | 23 | 0.0475 |
| GO.0051247 | positive regulation of protein metabolic process | 80 | 0.0478 |
| GO.0006950 | response to stress | 170 | 0.0487 |
| GO.0046330 | positive regulation of JNK cascade | 13 | 0.0498 |
