# Supplementary material for: Preeclampsia-Associated Alteration of DNA Methylation in Fetal Endothelial Progenitor Cells
Source: Front Cell Dev Biol. 2019 Mar 19;7:32. doi: 10.3389/fcell.2019.00032 (PMC6436196; doi:10.3389/fcell.2019.00032)
Supplement: TABLE S3 — List of KEGG pathways that were significantly enriched (FDR < 0.05) in passage 5 ECFC from preeclamptic patients versus passage 5 ECFC from healthy donors. [file Data_Sheet_3.PDF]

#pathway ID	pathway description	observed gen	false discovery rate
5205	Proteoglycans in cancer	37	0.00249
4310	Wnt signaling pathway	26	0.00283
4150	mTOR signaling pathway	15	0.00398
4510	Focal adhesion	33	0.00409
4520	Adherens junction	16	0.0054
5169	Epstein-Barr virus infection	30	0.0098
4144	Endocytosis	30	0.0111
4611	Platelet activation	22	0.0116
4915	Estrogen signaling pathway	18	0.0116
4152	AMPK signaling pathway	21	0.0121
4110	Cell cycle	21	0.0128
5202	Transcriptional misregulation in cancer	26	0.0128
5222	Small cell lung cancer	16	0.0186
5203	Viral carcinogenesis	27	0.0226
4728	Dopaminergic synapse	20	0.0305
4919	Thyroid hormone signaling pathway	19	0.0305
5100	Bacterial invasion of epithelial cells	14	0.0305
4921	Oxytocin signaling pathway	23	0.0327
4151	PI3K-Akt signaling pathway	42	0.0348
4390	Hippo signaling pathway	22	0.0373
5212	Pancreatic cancer	12	0.0373
4022	cGMP-PKG signaling pathway	23	0.0375
5162	Measles	20	0.0375
5223	Non-small cell lung cancer	11	0.0375
4261	Adrenergic signaling in cardiomyocytes	21	0.0422
4910	Insulin signaling pathway	20	0.0422
4725	Cholinergic synapse	17	0.0461
5206	MicroRNAs in cancer	21	0.0461
4010	MAPK signaling pathway	32	0.0481
4540	Gap junction	14	0.0481
4724	Glutamatergic synapse	17	0.0481
5166	HTLV-I infection	32	0.0481
5200	Pathways in cancer	39	0.0481
