# Supplementary material for: Preeclampsia-Associated Alteration of DNA Methylation in Fetal Endothelial Progenitor Cells
Source: Front Cell Dev Biol. 2019 Mar 19;7:32. doi: 10.3389/fcell.2019.00032 (PMC6436196; doi:10.3389/fcell.2019.00032)
Supplement: TABLE S4 — List of KEGG pathways that were significantly enriched (FDR < 0.05) in passage 3 ECFC from preeclamptic patients versus passage 3 ECFC from healthy donors. [file Data_Sheet_4.PDF]

#pathway ID	pathway description	observed gene count	false discovery rate
5203	Viral carcinogenesis	24	0.000402
5169	Epstein-Barr virus infection	24	0.000436
4144	Endocytosis	21	0.011
5205	Proteoglycans in cancer	22	0.0184
5202	Transcriptional misregulation in cancer	18	0.0189
4010	MAPK signaling pathway	23	0.0311
4915	Estrogen signaling pathway	12	0.0311
4310	Wnt signaling pathway	15	0.0316
4724	Glutamatergic synapse	13	0.0316
5206	MicroRNAs in cancer	15	0.0456
